# Predicting and affecting response to cancer therapy based on pathway-level biomarkers

**DOI:** 10.1038/s41467-020-17090-y

**Published:** 2020-07-03

**Authors:** Rotem Ben-Hamo, Adi Jacob Berger, Nancy Gavert, Mendy Miller, Guy Pines, Roni Oren, Eli Pikarsky, Cyril H. Benes, Tzahi Neuman, Yaara Zwang, Sol Efroni, Gad Getz, Ravid Straussman

**Affiliations:** 1 Department of Molecular Cell Biology, Weizmann Institute of Science, Rehovot, Israel; 2 Broad Institute of MIT and Harvard, Cambridge, MA 02145 USA; 3 Department of Thoracic Surgery, Kaplan Medical Center, Affiliated to the Hebrew University School of Medicine, Rehovot, Israel; 4 Department of Veterinary Resources, Weizmann Institute of Science, Rehovot, Israel; 5 Department of Pathology, Hebrew University of Jerusalem, Jerusalem, Israel; 6 The Mina and Everard Goodman Faculty of Life Sciences, Bar Ilan University, Ramat-Gan, 52900 Israel; 7 Massachusetts General Hospital Center for Cancer Research, Massachusetts General Hospital, Boston, MA 02114 USA; 8 Harvard Medical School, Boston, MA 02115 USA; 9 Department of Pathology, Massachusetts General Hospital, Boston, MA 02114 USA

**Keywords:** Cancer therapeutic resistance, Cellular signalling networks

## Abstract

Identifying robust, patient-specific, and predictive biomarkers presents a major obstacle in precision oncology. To optimize patient-specific therapeutic strategies, here we couple pathway knowledge with large-scale drug sensitivity, RNAi, and CRISPR-Cas9 screening data from 460 cell lines. Pathway activity levels are found to be strong predictive biomarkers for the essentiality of 15 proteins, including the essentiality of MAD2L1 in breast cancer patients with high BRCA-pathway activity. We also find strong predictive biomarkers for the sensitivity to 31 compounds, including BCL2 and microtubule inhibitors (MTIs). Lastly, we show that Bcl-xL inhibition can modulate the activity of a predictive biomarker pathway and re-sensitize lung cancer cells and tumors to MTI therapy. Overall, our results support the use of pathways in helping to achieve the goal of precision medicine by uncovering dozens of predictive biomarkers.

## Abstract (web summary)

Predicting an individual's response to therapy is an important goal for precision medicine. Here, the authors use an algorithm that takes into account the interaction type and directionality of signalling pathways in protein–protein interactions and find that their pathway analysis can predict essential genes, which may be a target for therapy.

## Introduction

The principle of precision medicine is to optimize medical care by tailoring the treatment of patients based on their genetic and molecular characteristics. To achieve this goal, it is necessary to discover and develop biomarkers that help decide which patients to treat (prognostic biomarkers) and which therapy is most likely to be effective (predictive biomarkers)^1,2^. However, identifying high-quality biomarkers that are accurate and robust across different experimental conditions is challenging.

For biomarkers that predict a cancer’s susceptibility to a drug, detection of either (i) the presence of somatic DNA alterations or (ii) specific gene expression patterns have been used. Of these detection methods, the most common has been detection of somatic DNA alterations in single genes (e.g., BRAF V600E mutations are associated with response to BRAF inhibitors in melanoma^3–5^), partly because measuring somatic mutations has less noise and batch effects compared to measuring gene expression^6–8^, and somatic events represent changes specific to cancer cells. Biomarkers based on gene expression levels, on the other hand, are less common because they are much more prone to batch effects, can vary greatly among different cell types, and are affected by the expression levels of genes in both cancer and non-cancer cells. Despite these challenges, a few expression-based biomarkers were shown to improve predictions beyond the traditional clinical and pathological parameters in various cancer types^9–13^. Most of these biomarkers were based on using expression levels across multiple genes or gene pathways rather than single-gene biomarkers because they more reliably and robustly predicted response^14–17^. Pathway-level methods were also more robust against batch effects, thus enabling the aggregation of data from various cohorts, resulting in highly significant biomarkers^18–22^. Historically, identifying pathway-based expression biomarkers was limited due to the lack of large-scale gene expression data from many cancer cell lines coupled with their sensitivity to a large cohort of anti-cancer drugs. However, the recent technological advances in gene silencing and editing enabled the generation of large-scale gene essentiality data (i.e., by CRISPR or RNA interference [RNAi]) in hundreds of cell lines derived from many different tumor types. This source of gene dependency data may enable the discovery of previously unknown altered pathways that are predictive of a tumor’s response to a drug. This can potentially uncover important biomarkers for several cancer types and treatments that are not possible to find at the single-gene expression level or through somatic DNA alterations.

In this study, we aimed to identify novel pathway-based predictive biomarkers across multiple tumor types for the response to different compounds by taking advantage of these recently available large datasets. To analyze this wealth of data, we chose to use a publicly available tool called PathOlogist^23^ that not only takes into consideration the specific protein–protein interactions in every pathway but also the interaction type and directionality of the signaling pathways (e.g., activation, inhibition). Indeed, accounting for the directionality of signaling pathways may increase both the accuracy and robustness of the analysis over other publicly available pathway-level tools that evaluate only gene sets. Here, we integrate gene expression and drug sensitivity datasets measured for hundreds of anti-cancer drugs across 460 cancer cell lines from 10 tissue types (breast, CNS, large intestine, lung (NSCLC and SCLC), ovary, pancreas, skin, stomach, and upper aerodigestive tract). To ensure robust findings, we use pathway activity levels generated from both microarray and RNA-seq data and tested their association with sensitivity measured in two different drug screening efforts, the Cancer Target Discovery and Development (CTD^2^)^24^ and Genomics of Drug Sensitivity in Cancer (GDSC)^25^. Furthermore, we (i) use highly conservative analytical methods and (ii) validate our predictive biomarkers using independent datasets as well as in patient-derived xenograft models (PDX) and fresh human tumors grown ex-vivo. Overall, our work illustrates the utility of identifying pathways whose activity levels can be used to predict, and potentially control, the response to specific compounds.

## Results

### Pathway activity levels are highly similar across platforms

The discovery of novel robust disease biomarkers is a major challenge. The reproducibility of such biomarkers across independent platforms and datasets is an essential requirement before they can become clinically relevant^15–17^. Previous studies suggest that pathway-based biomarkers may have a higher reproducibility than individual gene-based biomarkers^18,19^. This makes sense from both biological and statistical standpoints since biological processes often affect many genes simultaneously, offering the opportunity to aggregate readouts across many genes and thus extract a more stable metric from the “noisy” expression pattern of individual genes. While a common way to calculate a score for a pathway is to average the expression level of its member genes, here we employed the PathOlogist tool^23^, as it uses the structure of gene relationships within the pathway rather than treat the genes as simply a uniform set of entities. For each interaction node in a pathway, the PathOlogist tool calculates its potential to occur by determining the expression of its input proteins. An activity score of ‘1’ for an interaction indicates that all positively regulating proteins are being highly expressed, while inhibitory proteins are not expressed. The activity score of each of the pathways (ranging from 0 to 1) is then calculated by averaging the activity scores of all interactions within a pathway. A more detailed explanation of how pathway activity scores are being calculated can be found in the Supplementary Methods section as well as in Greenblum et al.^23^. Importantly, taking into account the structure of the pathway may have a major effect on the final pathway activity score as compared to averaging the genes’ expression levels. For example, the positive or negative regulation that each protein has on every interaction in the pathway is not reflected by averaging gene expression. Also, hub proteins that influence multiple interactions in a pathway will affect the PathOlogist activity scores of all of these interactions, as opposed to giving the same weight to all genes, if we simply average the expression of all genes in the pathway (Supplementary Fig. 1a).

**Fig. 1:**
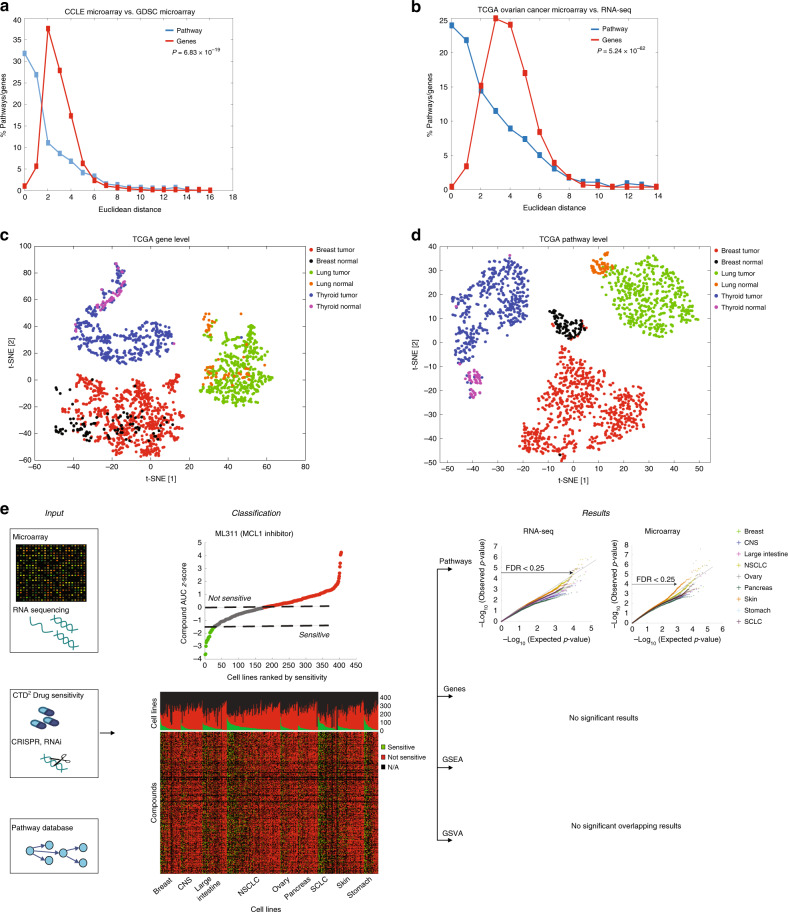
Pathway activity levels as predictive biomarkers. **a** Euclidean distance (ED) distribution of genes and pathways calculated from microarray data from two different institutions (CCLE and GDSC) across 438 cell lines. Blue line: ED distribution between the pathways in the two datasets; red line: ED distribution between the genes.* P-*values were generated using Mann–Whitney *U*-test. **b** ED distribution of genes and pathways between RNA-seq and microarray data across 294 ovarian cancer patients. Blue line: ED between the pathways; red line: ED between the genes. *P*-values were generated using Mann–Whitney *U*-test. **c** tSNE plot of the gene-expression levels in three tumor types and their adjacent normal tissue. Samples are colored by tissue type and state (tumor/normal). **d** tSNE plot of the pathway activity levels in three tumor types and their adjacent normal tissue. Samples are colored by tissue type and state (tumor/normal). **e** Workflow pipeline depicting the data flow from the (i) Input data to (ii) the drug-based Classification step to (iii) the final Results output. The quantile–quantile (QQ) plots are colored by tissue type. See also Supplementary Figs. 1–3.

Overall, we used PathOlogist to calculate the activity score of a total of 1028 curated pathways comprised of (i) the original 579 pathways already in the PathOlogist dataset including PID pathways^26^ (Biocarta, KEGG, and Nature/NCI) plus (ii) an additional 449 pathways that we added from PharmKGB^27^, Wikipathways^28^, and SignaLinks^29^ (see “Methods” section). In order to confirm the robustness of pathway-level activities, we evaluated the consistency of gene expression and pathway activities between experiments and platforms. We first took advantage of gene-expression microarray data that was generated for the same 438 cell lines at two different research centers: the GDSC project from the Wellcome Trust Sanger Institute and Massachusetts General Hospital^25,30^, and the Cancer Cell Line Encyclopedia (CCLE) project from the Broad Institute and Novartis^31,32^. To compare the stability of expression values of genes and the stability of pathway-level activities, we studied the distributions of Euclidean distances, a formal measure of similarity, between the corresponding values for each gene and pathway in these two datasets (see “Methods” section). Indeed, the distances were much lower for pathways than they were for individual genes (Fig. 1a), reflecting a higher similarity between the two platforms when pathway activity levels were used rather than single-gene expression. We further demonstrated that pathway-level analysis could also help reduce experimental platform effects by comparing RNA-Seq and microarray data from 294 human ovarian tumors from The Cancer Genome Atlas (TCGA; Fig. 1b).

To further validate the approach of pathway activity level scores calculated by PathOlogist, we tested whether these scores could better discriminate between normal and tumor states as well as among different tumor subtypes. We analyzed TCGA RNA-seq data from three different tumor types (breast, lung, and thyroid) that had a large number of tumor and tumor-adjacent normal samples. We used t-SNE dimensionality reduction^33^ to visualize the data and observed that, when using individual genes, the samples correctly partitioned according to the different tumor types but did not clearly distinguish between the normal and tumor states. In contrast, the t-SNE of pathway activities clearly separated the normal and cancer samples, as well as the different subtypes in breast cancer patients (Fig. 1c, d and Supplementary Fig. 1b, c). To evaluate the robustness of the clusters, we used three standard metrics (Silhouette^34^, Calinski–Harabasz^35^, and Dunn index^36^), for which higher values indicate more robust clustering (i.e., higher dissimilarity between clusters and tighter clusters). Here again, we observed that pathway activity levels cluster the samples more robustly (Supplementary Fig. 1d–f). To verify that the robust clustering based on pathway activities was not due to the different number of features used for clustering (number of pathways and genes), we performed the analysis using three sets of genes: (i) all 10,720 genes found in both microarray and RNA-seq datasets; (ii) 2512 genes from the 1028 pathways, (iii) and the 500 most variable genes. Indeed, pathway activity levels yielded more robust clusters compared to all the three sets of genes (Supplementary Fig. 1g–j). Furthermore, to test whether the biological meaning of the pathways (i.e., the specific genes in each pathway) are driving the signal, we randomized the pathway memberships by shuffling the gene identities but keeping the structure and number of genes in the pathway the same. We then calculated the Euclidean distance between the results in both cohorts and, as can be seen in Supplementary Fig. 1i, the distances are indeed random. These results show that random grouping of genes as pathways, without biological context, will not yield meaningful results.

### Using pathway activity levels to predict treatment response

To see the possible benefit of using pathway activity levels as biomarkers, and to see the differences between pathway-based biomarkers and single genes or gene sets biomarkers, we initially selected as input 460 cell lines from 10 cancer types for which we had available drug-response sensitivity data to 481 compounds from the CTD^2^ project. We additionally selected 254 and 307 cell lines for which the essentiality scores of more than 10,000 genes were tested by CRISPR or RNAi screens, respectively, in the Achilles project^37–39^. The association between activity levels and response was calculated within each tissue type separately to allow the detection of cancer type–specific biomarkers.

To prioritize predictive biomarkers that are robust across different platforms, we associated drug-response data with the pathway activity scores calculated using both RNA-Seq and microarray expression data of the cell lines. Cell line sensitivity to the compounds was classified by calculating *z*-score values derived from the area under the dose–response curve (dose–response AUC; dr-AUC; Supplementary Data 1). All cell lines with a z-score ≤ −1.5 were tagged as ‘sensitive,’ and all cell lines with *z*-score ≥ 0 were tagged as ‘not sensitive’ (see “Methods” section; Fig. 1e and Supplementary Fig. 1k–m). Next, we aimed to identify genes, gene sets, and pathways that were significantly associated with sensitivity of the cell lines to each of the drugs (using the Mann–Whitney *U*-test). We used (i) single-gene expression for 10,225 genes; (ii) GSEA (gene set enrichment analysis) with 50 hallmark and 4762 curated gene sets from the Molecular Signatures Database (MSigDB^40,41^); (iii) GSVA (gene set variation analysis) using the same gene sets as for GSEA, and (iv) PathOlogist pathway activity levels for 1028 pathways (Supplementary Data 2). Each of the ten cancer types were analyzed separately, and false-discovery rate (FDR) correction for multiple hypothesis testing^42^ was applied for each cancer type. We were unable to identify any MSigDB gene set score, either using GSEA or GSVA, that met significance (Fig. 1e and Supplementary Fig. 1k–m). However, using the PathOlogist activity scores, we identified 9 significant (*q* ≤ 0.05) and 31 near-significant (0.05 < *q* ≤ 0.25) pathways in both microarray and RNA-seq datasets. Within those identified, 25 pathways overlapped between the two platforms (Supplementary Information). We further validated these results using independent datasets (examples in Supplementary Fig. 2). Finally, to test if differences in numeracy between pathway counts and gene counts affect the number of significant results, we performed sensitivity and power analysis (see “Methods” section). Results of these analyses show that in both types of cell lines (NSCLC and skin melanoma), the number of significant pathways (*q* < 0.25) increased with the number of cell lines analyzed (Supplementary Fig. 4a, b), suggesting the importance of analyzing a large number of cell lines per tumor type (at least 30) for discovering significant tumor type-specific pathways.

### Pathways predict response to BRAF/MEK and EGFR inhibitors

Two pathways predicted the response to BRAF and MEK inhibitors in melanoma cell lines: the CREB and NFAT pathways (Supplementary Figs. 2 and 3). The presence of a *BRAF*^*V600E*^ mutation is a known predictive biomarker for the sensitivity of melanoma cells to BRAF and MEK inhibition^43,44^. As expected, we found that the activity level of both pathways were highly correlated with the presence of the *BRAF*^*V600E*^ mutation in the cell lines (note that *BRAF* and *MEK* are not part of these pathways, Supplementary Fig. 2a). Interestingly, it has been suggested that a CREB-dependent mechanism may cause resistance to BRAF–MEK inhibition in some melanoma patients^43^ and that NFAT is activated by oncogenic *BRAF*^*V600E*^ via canonical MEK/ERK signaling in melanoma cell lines^44^. These results are thus consistent with our observation that the CREB and NFAT pathways are highly significant and robust biomarkers for predicting the response to BRAF/MEK inhibitors. In addition, we also identified the activity of ‘MAPK inactivation of SMRT corepressor pathway’ as a good predictive biomarker for the sensitivity of EGFR-activated NSCLC cell lines to EGFR inhibitors. As expected, this pathway correlated with the presence of activating mutations in *EGFR* (Supplementary Fig. 4c, d).

### IL2–STAT5 pathway predicts response to BCL2 inhibitors

Cancer cells frequently adopt anti-apoptotic mechanisms that help them survive internal and external signals that initiate pro-apoptotic signaling. While excellent inhibitors were developed over the years to block the anti-apoptotic defense mechanisms in cancer cells (e.g., BCL-2 and Bcl-xL inhibitors), lack of specific predictive biomarkers for their use has limited their utility. Therefore, there is a clear unmet need for predictive biomarkers for these drugs^45^.

**Fig. 2:**
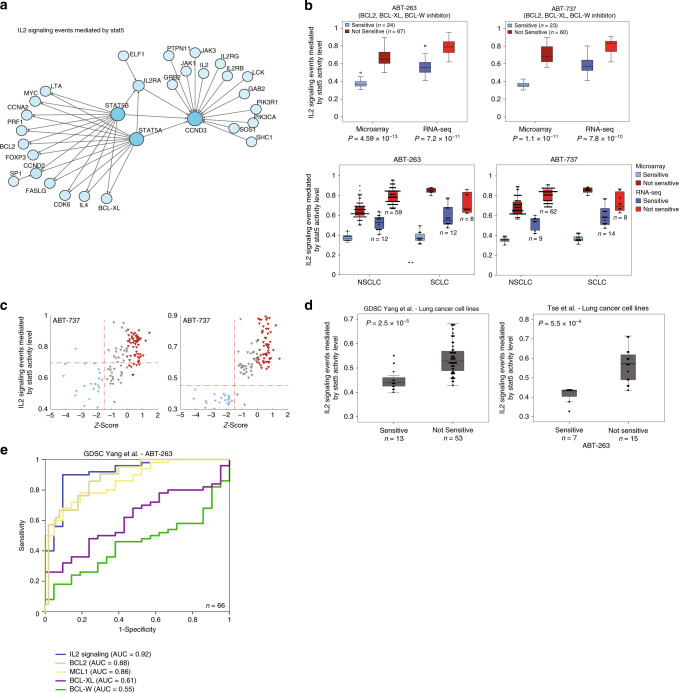
IL2–STAT5 pathway predicts response to BCL2 inhibitors. **a** Network diagram representing the *‘*IL2 signaling events mediated by STAT5′ pathway. **b** IL2–STAT5 pathway activity levels in sensitive and not-sensitive lung cancer cell lines in microarray and RNA-seq. Upper box plots represent all lung cancer cell lines, and lower box plots represent the SCLC and NSCLC subtypes. Error bars represent the standard deviation. *P*-values were generated using Mann–Whitney *U*-test. **c** Scatter plot of ABT-737 AUC *z*-score values versus IL2–STAT5 pathway levels in microarray and RNA-seq (blue: sensitive cell lines; red: not-sensitive cell lines; gray: samples that were excluded from the analysis). **d** Two independent datasets of lung cancer cell lines treated with ABT-263. Box plots show IL2–STAT5 pathway activity levels in sensitive and not-sensitive cell lines. Error bars represent the standard deviation. *P*-values were generated using Mann–Whitney *U*-test. **e** ROC analysis was constructed to evaluate the prognostic power of the IL2–STAT5 pathway versus ABT-263 targets and MCL1 in the validation set. The AUC was used to quantify response prediction. See also Supplementary Fig. 4.

Here, we identified the activity of the ‘IL2 signaling events mediated by STAT5’ pathway (Fig. 2a) as a robust biomarker for the response to two highly similar Bcl-2 protein–family inhibitors (ABT-263 and ABT-737) in lung cancer cell lines. The activation of STAT5 proteins (STAT5a and STAT5b) is one of the earliest signaling events downstream of the IL-2 cytokine and other IL-2 family members. This allows signals to quickly traverse from the membrane into the nucleus^46^. In the nucleus, activated STAT5 dimers bind to specific DNA-response elements located in the promoters of target genes to regulate various cellular responses, including growth and survival^47^. STAT5 is constitutively activated in several solid tumors, including prostate cancer^48^, breast cancer^49^, nasopharyngeal carcinoma^50^, and head and neck squamous cell carcinoma^51^. However, the precise role of STAT5 in epithelial carcinogenesis remains incompletely understood.

The ‘IL2–STAT5 signaling pathway’ contains 30 genes (including *BCL2* and *BCL2L1)* whose expression is directly induced by STAT5. Low pathway levels were significantly associated with sensitivity to ABT-263 and ABT-737 in both small-cell (SCLC) and non-small-cell lung cancer (NSCLC) cell lines but not in any other cancer type, while high pathway activity levels were associated with lack of sensitivity (Fig. 2b, c and Supplementary Fig. 4e, f). This association was validated in two independent datasets: the (i) GDSC drug-response and (ii) NSCLC cell line^52^ datasets (Fig. 2d). While the IL2–STAT5 signaling pathway activity level correlated well with responses to inhibitors of the Bcl-2 family of proteins, we did not observe a high correlation with any of the individual gene components of the pathway (Supplementary Figs. 4g and 5a). Moreover, the AUC calculated from receiver operating characteristic (ROC) curve analysis from both microarray and RNA-seq data was higher for IL2–STAT5 signaling activity than for *BCL2*, *BCL2L1*, and *BCL2L2* individual gene expression levels, which are all targets of ABT-263 and ABT-737 (Fig. 2e and Supplementary Fig. 5b) along with *MCL1*, a gene that when overexpressed is known to confer resistance to these compounds^53^. Interestingly, analysis of IL2–STAT5 signaling levels of NSCLC patients from TCGA showed high variability in pathway activity levels, suggesting that a sub-population of patients may benefit from treatment with BCL2 inhibitors such as ABT-263 and ABT-737 (Supplementary Fig. 5c). Collectively, these data suggest that IL2–STAT5 pathway activity can predict cellular sensitivity to Bcl-2 inhibitors in lung cancer with higher specificity and sensitivity than evaluating single genes in the Bcl-2 family alone.

### AIF pathway predicts lung cancer response to MTIs

**Fig. 3:**
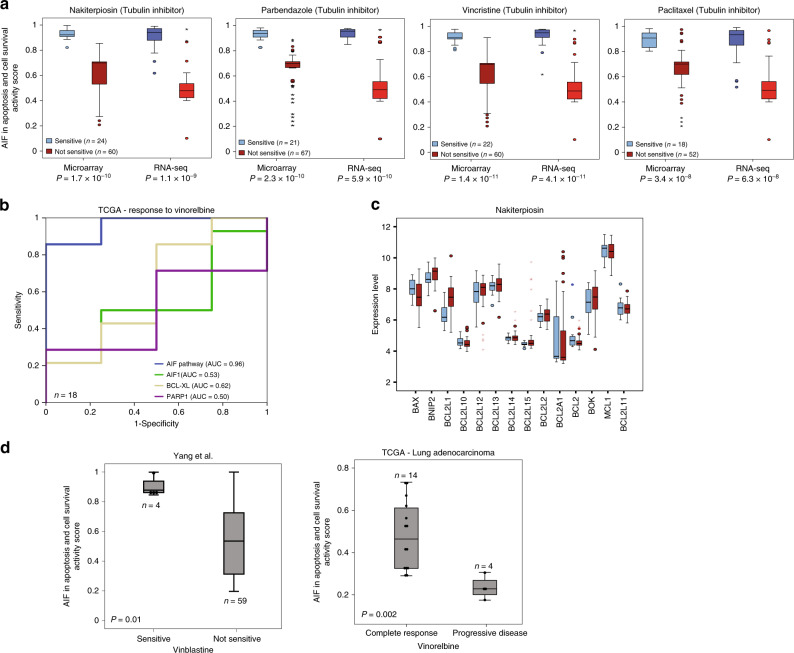
AIF in apoptosis and cell-survival pathway predicts response to MTI. **a** ‘AIF in apoptosis and cell-survival pathway’ activity levels in sensitive (blue) and not-sensitive (red) lung cancer cell lines in microarray and RNA-seq. Box plots represent the MTIs that were identified by this analysis across all lung cancer cell lines. Error bars represent the standard deviation. *P*-values were generated using Mann–Whitney *U*-test. **b** ROC analysis was constructed to evaluate the prognostic power of the AIF pathway versus the three pathway genes (*AIF1*, *BCL-XL*, *PARP1*) in the TCGA lung adenocarcinoma dataset. The AUC was used to quantify response prediction. **c** Box plot of BCL2-protein family member expression levels in sensitive (blue boxes) and not-sensitive (red boxes) cell lines. Error bars represent the standard deviation. **d** Validation sets of lung cancer cell lines^25^ and patients (TCGA) that were treated with MTIs. Box plots show AIF pathway activity levels in the sensitive and not-sensitive cell lines (left panel) as well as in patients with complete response or progressive disease (right panel). Error bars represent the standard deviation. *P*-values were generated using Mann–Whitney *U*-test. See also Supplementary Figs. 5, 6.

Microtubules (MTs) are important for many cellular functions involving the cytoskeleton^54–57^. Because of their essential role in cell division, MT inhibitors (MTIs) are used in the treatment of many solid and hematologic malignancies^58,59^. Here, we identified the activity of the apoptosis-inducing factor (AIF) pathway as a strong predictor for response to four different MTIs in both SCLC and NSCLC (Fig. 3a, Supplementary Figs. 5d, e and 6a, b). The AIF pathway is composed of three genes—*PARP1*, *BCL2*, and *BCL2L1*. AIF1 is a mitochondrial intermembrane flavoprotein that translocates to the nucleus in response to pro-apoptotic stimuli, such as initiated by PARP1, and induces nuclear apoptosis. The anti-apoptotic proteins, Bcl-2 and Bcl-xL (encoded by *BCL2L1*), block AIF1 translocation by directly sequestering members of the pro-apoptotic proteins like Bax. Interestingly, MTIs were shown to promote the release of Bax from Bcl-xL by inducing phosphorylation of Bcl-xL^60^. We reason that the high activity level of the AIF pathway reflects the pro-apoptotic pull in the delicate balance between anti- and pro-apoptotic forces. Treating cells that have high AIF pathway activity with MTIs may cause a drastic shift in this delicate balance by releasing large amounts of sequestered Bax proteins and tipping the balance into apoptotic cell death.

Interestingly, the expression levels of each of the three genes in the AIF pathway poorly classified the lung cancer cell lines as responders or non-responders to MTI (Supplementary Fig. 6c). However, the AIF pathway activity levels better predicted cellular response to MTIs than any of the single genes in the pathway (Fig. 3b). In fact, none of the known 14 Bcl-2 family-related genes was significantly correlated with MTI response (Fig. 3c and Supplementary Fig. 6e), demonstrating again that integrating the signals at the pathway level can significantly improve the classification of drug-response sensitivity compared to single genes. Of note, while we screened 10 apoptosis and cell survival-related pathways, only the AIF pathway could predict MTI response in a statistically significant manner (Supplementary Fig. 6d). Importantly, we found that the activity level in the AIF pathway correlated significantly with the response to MTIs in additional unrelated datasets (Fig. 3d). In the GDSC database, only 4 of the 63 tested lung cancer cell lines were sensitive to vinblastine, a clinically used MTI, and the average activity levels for the AIF pathway in these 4 responding cell lines were significantly higher than the average activity levels of the non-responding cell lines. Additionally, in the TCGA dataset, we identified 16 lung adenocarcinoma patients with a documented response to vinorelbine, another clinically used MTI. Patients exhibiting complete response had much higher AIF pathway levels than patients with progressive disease. Taken together, these data demonstrate that the AIF pathway level is a strong predictive biomarker for the response of lung cancer cells to MTIs.

### Increasing the AIF pathway sensitizes lung cancer to MTIs

**Fig. 4:**
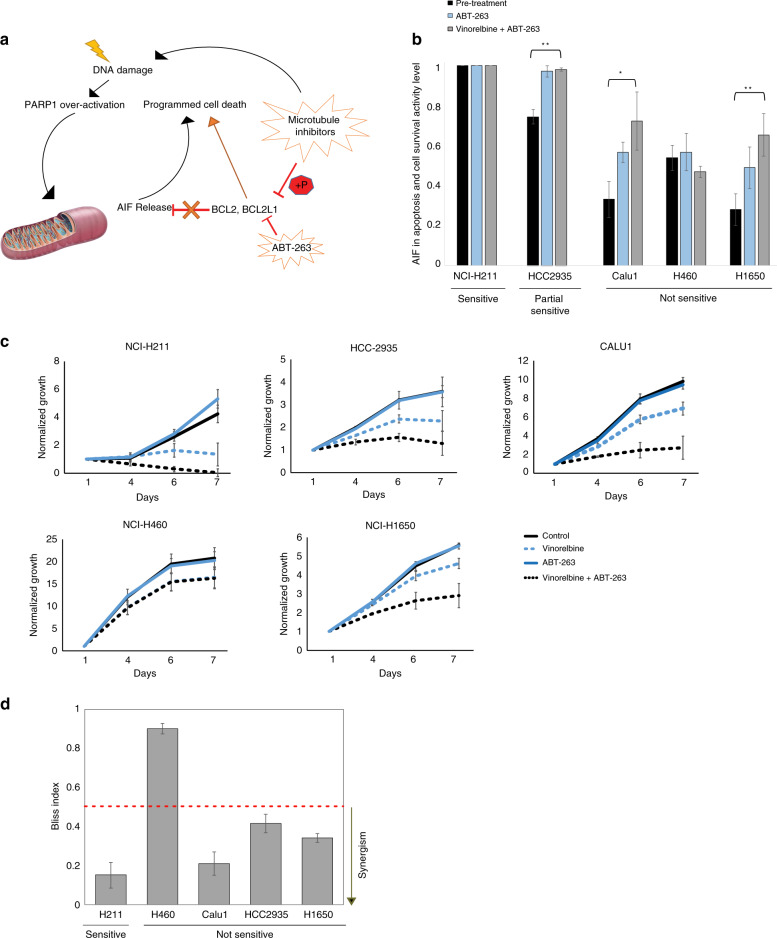
BCL-XL inhibition elevates AIF pathway levels resulting in a synergistic activity with MTIs. **a** Cartoon depicting the ‘AIF in apoptosis and cell-survival pathway’ with ABT-263 and MTI activity. **b** AIF pathway activity levels calculated from qRT-PCR in lung cancer cell lines before treatment (black bars), after treatment with ABT-263 (blue bars), and after treatment with a combination of vinorelbine and ABT-263 (gray bars). H211 was identified as sensitive, and the other four cell lines as not sensitive. Error bars represent the standard deviation. *P*-values were generated using Mann–Whitney *U*-test. **c** Relative growth of lung cancer cell lines over a 7-day period with no treatment (solid black line), vinorelbine (dotted blue line), ABT-263 (solid blue line), and a combination of vinorelbine and ABT-263 (dotted black line). Error bars represent the standard deviation. **d** Bliss analysis of drug synergy in cell lines treated with vinorelbine plus ABT-263. Bliss index < 0.5 represents synergism. Error bars represent the standard deviation. See also Supplementary Fig. 7a.

Thus far, our results have suggested that different pathway levels can predict response to specific therapies in various tissue types. We next sought to determine whether we could alter drug responses in cancer cells by modulating the activity levels of a predictive pathway. Specifically, we tested whether increasing the activity levels of the AIF pathway (Fig. 4a) in lung cancer cells could, as a consequence, increase their sensitivity to MTIs. As a significant negative correlation was observed between AIF pathway activity and the mRNA expression of Bcl-xL (Supplementary Fig. 7a), we hypothesized that targeting Bcl-xL could increase AIF pathway activity levels and induce cellular sensitivity to MTIs. We first selected three lung cancer cell lines from the CTD^2^ database that were resistant to vincristine (H460, Calu1, and H1650), one partially resistant cell line (HCC2935), and one cell line that was highly sensitive (H211). We measured the expression levels of the pathway genes using RT-qPCR and found, as expected, that the sensitive cell line H211 had a higher AIF pathway activity level than the four resistant cell lines (Fig. 4b). We then treated all five cell lines with ABT-263 and found that it increased AIF pathway activity levels in three out of the four resistant cell lines (Fig. 4b); only H460 did not alter the AIF pathway activity. In addition, co-treatment of the cell lines with both ABT-263 and vinorelbine resulted in comparable or higher activation of the AIF pathway in the same three cell lines, as measured 24 h after treatment, but again not in H460.

To assess whether increasing AIF pathway activity levels could sensitize cancer cells to MTIs, we treated the cell lines with vinorelbine together with ABT-263 or DMSO control for 7 days. In the three cell lines where ABT-263 increased AIF pathway levels, we observed significantly increased sensitivity to vinorelbine. In contrast, the ABT-263–treated H460 cell line remained insensitive to vinorelbine, a result consistent with the inability of ABT-263 to alter AIF-pathway activity in this particular cell line (Fig. 4c). Bliss independence analysis confirmed that ABT-263 did synergize with vinorelbine in four out of the five cell lines (Fig. 4d). Thus, our results suggest that the identified AIF pathway not only correlates with and predicts drug sensitivity but also has a causal role, wherein altering the AIF pathway can modulate cellular response to MTIs. Interestingly, while this is the first time that a synergistic effect between MTIs and Bcl-xL inhibition has been demonstrated in lung cancer, a synergistic activity between these drugs was also demonstrated in other cancer types^60^.

**Fig. 5:**
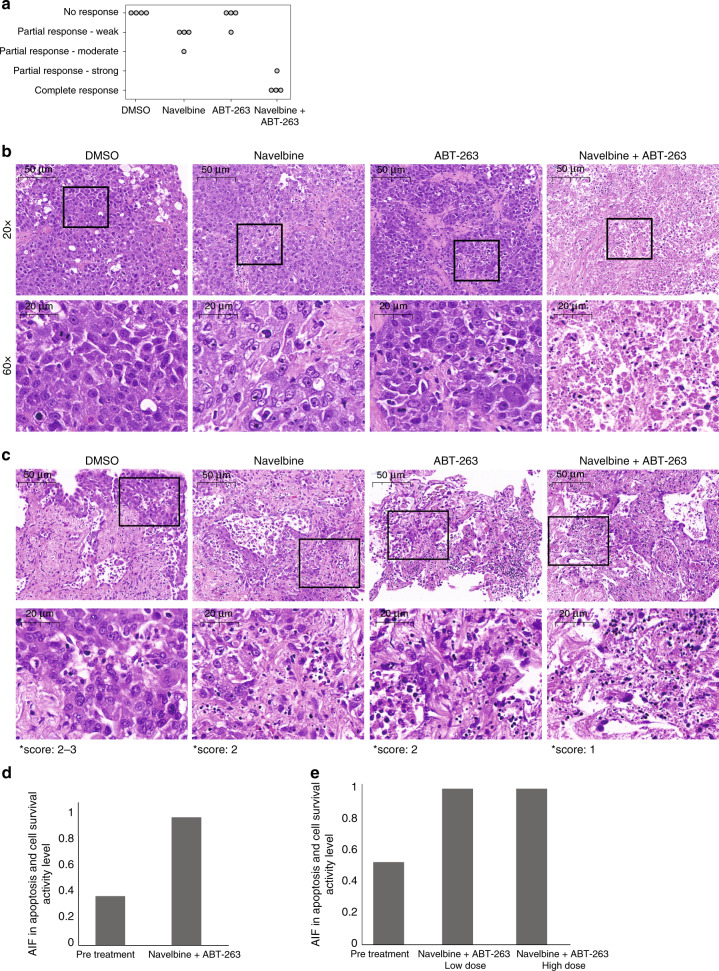
Synergistic activity of ABT-263 and Navelbine in PDX-model and human lung cancer patient. **a** Cell viability in ex-vivo organ culture. Viability values are as follows: 0: 0–20% viability, complete response; 1: 20–35%, partial response: strong; 2: 36–59%, partial response: moderate; 3: 60–84%, partial response: weak; 4: 85–100%, no response. Viability percentages were quantified by evaluating morphological features and were performed by two pathologists that were blinded to the experiment. **b** H&E-stained histology of NSCLC PDX tumors treated ex-vivo with DMSO (control), Navelbine, ABT-263, and a combination of the two drugs. **c** H&E-stained histology of a tumor from an untreated 71-year-old patient. The samples treated ex-vivo with DMSO (control), Navelbine, ABT-263, and a combination of the two drugs. **d** AIF pathway activity levels calculated from qRT-PCR in FFPE-preserved tissues from (**b**). *P*-values were generated using Mann–Whitney *U*-test. **e** AIF pathway activity levels calculated from qRT-PCR in FFPE-preserved tissues from (**c**) before and after treatment with a combination of navelbine and ABT-263. *P*-values were generated using Mann–Whitney *U*-test. See also Supplementary Fig. 7b.

To further demonstrate the synergistic activity of ABT-263 and MTIs in human lung cancer, we used a NSCLC patient-derived xenograft (PDX) model. PDX tumors were removed and sectioned into 250 μM slices (see “Methods” section) and then treated ex vivo with (i) DMSO, (ii) Navelbine (vinorelbine), (iii) ABT-263, or (iv) a combination of the two drugs. Four biological repeats were carried out, each using four PDX tumors, and tissue viability was assessed by H&E staining (Fig. 5a, b). In agreement with our in-silico and in-vitro results, we found that while Navelbine and ABT-263 only weakly affected the tumors when administered alone, a complete or near-complete response was observed in all PDX tumors that were treated with the drug combination (Fig. 5b). In addition, an increase in apoptosis was observed when treating the tumors with the combined treatment, as determined by cleaved caspase-3 staining (Supplementary Fig. 7b). Furthermore, a fresh tumor from a 71-year-old treatment-naive NSCLC patient was treated ex vivo as described above. Tissue viability was assessed and scored using the Modified Ryan Scheme for tumor regression score. Once again, the combined treatment of ABT-263 and Navelbine had a larger effect on response (Fig. 5c) as compared to the administration of either ABT-263 or Navelbine alone. RNA was extracted from the FFPE blocks before and after treatment, and qRT-PCR was then performed in order to calculate the pathway activity levels (Fig. 5d, e). We found that AIF pathway activity levels were low before treatment and then increased after being treated with the drug combination both in PDX-mice and in the fresh human tumor, supporting our previous results (Fig. 4).

Taken together, our results highlight (i) the sensitivity of lung cancer cells with high activity of the AIF-apoptosis pathway to MTIs, and (ii) the synergistic effect of Bcl-xL and MTIs in lung tumors with initial low activity level of the AIF-apoptosis pathway.

### Signaling pathway levels predict gene essentiality in cancer

Over the last decade, multiple genome-wide functional genomic screens identified genes that were essential for the proliferation and survival of cancer cells^61–67^. While specific inhibitors are available for some of these targets, many others are still lacking clinical-grade small-molecule inhibitors. The presence of predictive biomarkers for the essentiality of specific genes may prioritize the development of such small molecules or RNAi interventions that can target these genes.

To test whether pathway activity scores can serve as predictive biomarkers for the essentiality of genes, we used genome-wide RNAi and CRISPR screens from the Achilles project testing gene essentiality. Overall, we were able to find pathway activity levels that significantly predicted the essentiality of 16 genes (Supplementary Information). For example, the ‘IL2 signaling events mediated by PI3K’ pathway (*n*_genes_ = 32; Supplementary Fig. 8a) could predict the essentiality of *BRAF* in melanoma cell lines (Supplementary Fig. 8b). Interestingly, we found that low pathway activity levels were associated with sensitivity and overlapped with the *BRAF*^*V600E*^ mutation (Supplementary Fig. 8c–e), which is known to correlate with sensitivity to inhibition of BRAF activity^68^. Two of the novel biomarkers of gene essentiality that we found are highlighted below.

### Stathmin pathway predicts NSCLC sensitivity to CLTC RNAi

Clathrin heavy chain 1 (CLTC) is an important component of the cytoplasmic face of intracellular organelles. CLTC also plays an important role in mitotic spindle assembly and chromosome congression in the metaphase plate through a microtubule-mediated mechanism^69^. It colocalizes with β-tubulin (TUBB) at the mitotic spindle, and the appearance and disappearance of CLTC along with TUBB occurs during spindle activity^70^. Inhibition of CLTC was previously shown to induce apoptosis in colon cancer cell lines by disrupting bipolar spindle formation^71^. As such, it may play an important role in determining cellular sensitivity to chemotherapeutic agents^72^.

**Fig. 6:**
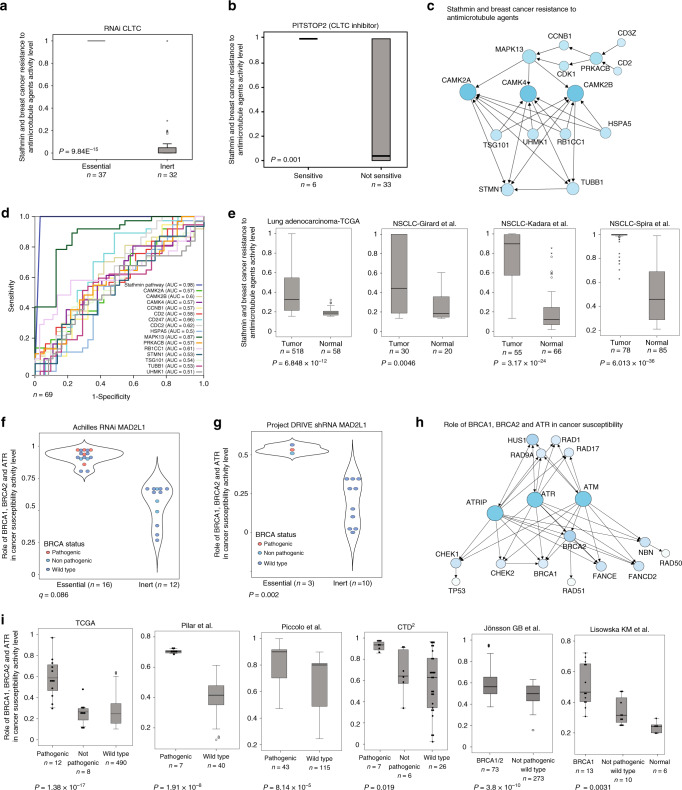
Pathways predict gene essentiality. **a** ‘Stathmin resistance to anti-microtubule’ pathway activity levels in *CLTC*-essential and -inert NSCLC cell lines. Error bars represent the standard deviation. *P*-values were generated using Mann–Whitney *U*-test. **b** Stathmin pathway activity levels in sensitive and not-sensitive NSCLC cell lines to PITSTOP2 (CLTC inhibitor). **c** Network diagram representing the *‘*Stathmin resistance to anti-microtubule*’* pathway. **d** ROC analysis was constructed to evaluate the prognostic power of the Stathmin pathway versus the 13 pathway genes and *CLTC*. The AUC was used to quantify response prediction. **e** Box plots of Stathmin pathway activity levels in NSCLC tumor samples and their adjacent normal tissues in four independent datasets. Error bars represent the standard deviation. *P*-values were generated using Mann–Whitney *U*-test. **f** Violin plot of ‘Role of BRCA1, BRCA2, and ATR in cancer susceptibility’ pathway activity levels in *MAD2L1* essential and inert breast cancer cell lines from the Achilles project. Dots are colored by BRCA1/2 mutation status. *P*-values were generated using Mann–Whitney *U*-test. **g** BRCA pathway activity levels in MAD2L1 essential and inert breast cancer cell lines from project DRIVE. Dots are colored by BRCA1/2 mutation status. *P*-values were generated using Mann–Whitney *U*-test. **h** Network diagram representing the ‘Role of BRCA1, BRCA2, and ATR in cancer susceptibility’ pathway. *P*-values were generated using Mann–Whitney *U*-test. **i** BRCA pathway activity levels in breast cancer patients with pathogenic, non-pathogenic, or wild-type *BRCA1/2* mutation in six independent cohorts. Error bars represent the standard deviation. *P*-values were generated using Mann–Whitney U-test. See also Supplementary Fig. 8.

Here, we show that elevated stathmin pathway activity level is highly associated with sensitivity to *CLTC* inhibition by RNAi (Fig. 6a) in NSCLC cell lines. In agreement with this observation, we found that the sensitivity of NSCLC cell lines to the CLTC inhibitor PITSTOP2 also correlated with stathmin pathway activity (Fig. 6b). We found that the stathmin pathway (*n*_genes_ = 15, Fig. 6c) is either always on (large intestine, pancreas, and stomach) or always off (CNS and skin), with variability only observed in lung, ovary, and breast (partial variability) tissues (Supplementary Fig. 8f). Nevertheless, this pathway only had statistical power to predict *CLTC* essentiality in NSCLC. When comparing the ability of the stathmin pathway to predict sensitivity to CLTC inhibition with single genes, we found that while a few single genes in the stathmin pathway did also correlate with the essentiality of *CLTC* in NSCLC cell lines, the stathmin pathway activity score consistently exhibited the highest correlation and area under the ROC curve (Fig. 6d and Supplementary Fig. 8g).

Interestingly, we found that stathmin pathway levels were highly elevated in NSCLC tumors as compared to normal lung tissues in four different NSCLC datasets^73,74^ (GSE31547 unpublished) and in > 500 TCGA patients (Fig. 6e). Although normal lung tissue contains a mix of different cell types and not just the epithelial cells that give rise to NSCLC, this finding may imply the existence of a therapeutic window for CLTC inhibition in lung cancer. Taken together, these results suggest that the activity of the stathmin pathway may serve as a predictive biomarker for the essentiality of the *CLTC* gene in NSCLC.

### BRCA pathway predicts MAD2L1 essentiality in breast cancer

Approximately 12% of women in the general population will develop breast cancer during their lives^75^. In contrast, approximately 72% of women who inherit a pathogenic *BRCA1* mutation and 69% of women who inherit a pathogenic *BRCA2* mutation will develop breast cancer by the age of 80. While breast cancer tumors with *BRCA* mutations are more susceptible to treatment with PARP1 inhibitors, there is still a great clinical need for novel drugs that can be used to treat advanced *BRCA*-mutated breast cancer.

Here, we found that high activity levels of the pathway ‘Role of BRCA1, BRCA2, and ATR in cancer susceptibility’ (BRCA pathway; *n*_genes_ = 17 , Fig. 6h) were highly associated with *MAD2L1* essentiality in breast cancer cell lines (Fig. 6f). This result was validated using data from Project DRIVE^76^, a genome-wide shRNA screen from Novartis (Fig. 6g). The BRCA pathway is composed of genes upstream of *BRCA1/2* that control cell cycle, DNA repair, cell division, and cell death (Fig. 6h). In particular, *MAD2L1* is an essential cell cycle-associated protein that functions as a spindle assembly checkpoint protein that prevents the onset of anaphase until all chromosomes are properly aligned at the metaphase plate^77^. *MAD2L1* overexpression leads to chromosomal instability in lung cancer and short survival^78^, and has also been associated with early metastasis in breast cancer^79^.

Five breast cancer cell lines were positive for pathogenic *BRCA1/2* mutations. Interestingly, all of them were part of the *MAD2L1*-essential group (Fig. 6f), implying a possible connection between the essentially of MAD2L1 and *BRCA1/2* mutational status.

While *BRCA1* and *BRCA2* expression levels themselves cannot predict the presence of pathogenic mutations in these genes (Supplementary Fig. 8h), BRCA pathway levels highly correlate with the presence of pathogenic mutations in these genes. Indeed, we found a significant association between the presence of *BRCA1/2* pathogenic mutations and high BRCA pathway levels in 6 different breast cancer datasets (Fig. 6i). Accordingly, ROC curve analysis demonstrated the high predictive power of BRCA pathway activity levels of *BRCA1/2* pathogenic mutations as compared to any single gene in the pathway (Supplementary Fig. 8i).

Overall, these results uncover a potential link between *MAD2L1*, *BRCA1/2* pathogenic mutations, and high levels of BRCA pathway activity. Our results point to a subpopulation of cell lines that have a high activity level of the BRCA pathway despite the absence of a known *BRCA1/2* pathogenic mutation. This may highlight the potential of *MAD2L1* to act as a possible therapeutic target not only in *BRCA1/2*-mutated breast cancer but also in a wider group of patients with a high activity level of this pathway.

## Discussion

In order to select the best drug treatment strategy for a given individual tumor, better predictive biomarkers of tumor response are clearly needed, but finding accurate and robust biomarkers has proven to be a challenge due to underpowered studies and the use of single genes or gene sets with only a few genes.

One effort to identify and benchmark methods for predicting therapeutic response is the NCI-DREAM drug sensitivity prediction challenge^80^. This effort identified the top-performing approaches as those that modeled nonlinear relationships and incorporated biological pathway information. Furthermore, gene expression consistently provided the best predictive power compared to other genetic or epigenetic datasets. A second effort for inferring genetic predictors of gene essentialities^81^ also found that gene expression was the most informative molecular data type. However, as described in these two efforts, integration of additional genetic and epigenetic data types can improve the overall predictive power. Here, we used knowledge about protein–protein interactions within pathways and gene expression data, but as suggested by these two efforts, future integration of additional data types might enhance the performance of our predictions.

By incorporating multiple available novel datasets and tools that take into account the combined influence of different components of a pathway, we were able to find a large number of novel biomarkers as well as the following other important advances in discovering biomarkers based on pathway-based analyses: (1) our results demonstrate that relatively large cohorts of cell lines from each cancer type are needed in order to be powered enough to detect statistically significant biomarker pathways (Supplementary Fig. 4a, b). Indeed, we made use of relatively recent datasets measuring both the expression pattern of hundreds of human cancer cell lines and the sensitivity of the same cell lines to hundreds of drugs, or to genome-wide gene targeting by both shRNA and CRISPR. (2) We chose the PathOlogist tool that takes into account prior knowledge about the structure of each pathway to calculate the pathway activity scores and enhanced the functionality of this tool by extending its database of pathways. (3) By taking a highly conservative approach for defining significant associations to minimize false positives results, we were able to validate all our findings in unrelated datasets and a few chosen ones by experimental follow up. Thus, our findings add new validated biomarkers to the list of previously defined pathway biomarkers for predicting drug response in the literature, such as (i) a five-gene hedgehog signature that can identify Hedgehog-activated tumors in medulloblastoma^82^ and (ii) a p53 signature that can refine the selection of patients that will likely respond to p53–MDM2 inhibitors^83^.

One can consider biomarkers that are based on pathway-level activities as a way of combining and improving the performance of multiple gene-level biomarkers, and thus increasing the confidence and accuracy of therapeutic decision-making in the clinic^82^. Importantly, biomarkers can provide guidance on which patients to treat, or not treat, with a specific drug. It is also possible that pathway-level biomarkers may turn out to be more powerful exclusion, rather than inclusion, criteria for some therapies.

RNAi therapy is an emerging approach for achieving precision cancer therapy^83^ and could potentially be used to follow-up on biomarkers found by pathway analysis. Since our data show that the analysis of pathways can predict biological responses, it is reasonable to consider that this type of analysis will yield good candidate pathways for RNAi therapy. Our analysis of RNAi and CRISPR-Cas9 screens identified novel genes in multiple tissue types that may not only serve as predictive biomarkers for response to different drugs but also as potential therapeutic targets.

It is important to note that one limitation of our biomarker discovery approach is that the activity of pathways is not always regulated, or at least not always reflected, by mRNA abundance. Indeed, regulation of protein translation and different post-translational modifications (e.g., protein phosphorylation) can commonly affect protein activity levels and the resulting pathway activities. For pathways in which the activity level is not reflected by changes in the expression patterns of the pathways’ members, our analysis will be underpowered and likely miss a potential association with response, resulting in false-negative results. We speculate that the activity of pathways controlled by transcription factors may be easier to detect by gene expression data. Nevertheless, even classic signaling pathways that propel the signaling flux along the pathway via post-translational modification events commonly affect downstream transcriptional activity, and thus may also be detected by mRNA expression data.

It should also be noted that while the PathOlogist tool takes into account prior data about protein–protein interactions, as well as the interaction type and directionality, these connections can change among different tissues and cancer types. Future availability of tissue-specific connections may result in even better and more accurate predictive biomarkers. Still, our ability to find and validate multiple novel biomarkers speaks to the fact that, at least in some, the pathway expression data coupled with prior knowledge of the interactions between the proteins in the pathway and their directionality is reflective of the pathway’s activity.

In summary, personalized predictive biomarkers can help us identify which tumors will be sensitive to a certain drug. The work presented here provides evidence that our novel, stringent methods can confidently identify pathways that can serve as robust and powerful biomarkers. Based on our findings, which we validated in several independent datasets and in human-derived cell lines and tumors, we propose a way of identifying new and clinically relevant therapeutic combinations that take into acccount a patient’s specific molecular expression pattern. Finally, based on our sensitivity analyses, we observe a rapid increase in the number of pathway-level biomarkers with the number of cell lines of a given tumor type (or subtype); hence, we expect additional biomarkers to be found with larger drug-sensitivity and gene-dependency datasets.

## Methods

### Cell lines

All cells were maintained in Dulbecco’s Modified Eagle’s Medium (DMEM) (Invitrogen, #10569-010) containing 10% fetal bovine serum (FBS) and 1% penicillin–streptomycin with glutamine (Invitrogen, #15140-122). For green fluorescent protein (GFP) expression in the cancer cell lines, lentiviral transduction was carried out using the pLex_TRC206-GFP plasmid.

### Ex-vivo organ culture

Mouse experiments were performed in accordance with the Institutional Animal Care and Use Committee (IUCUC) guidelines. Patient-derived xenograft (PDX) models were purchased (The Jackson Laboratories, model number TM00204) by the Weizmann Institute and licensed for institutional use.

Mice were euthanized with CO_2_, and the tumor tissues were removed and placed in ice-cold PBS. Tumors were sliced to 250-μM thick slices using a vibratome (VF300, Precisionary Instruments, Boston USA), placed in 6-well plates on titanium grids (Alabama R&D, Munford, USA) with 4 mL of DMEM/F12 medium (supplemented with 5% FCS, penicillin 100 IU/mL with streptomycin 100 μg/mL, amphotericin B 2.5 μg/mL, gentamicin sulfate 50 mg/mL, and L-glutamine 100 μL/mL). Tissue was cultured at 37 °C, 5% CO_2_, and 80% O_2_, on an orbital shaker (TOU-120N, MRC) at 70 rpm. The following day, tissue was treated with drugs as indicated for 96 h, followed by formalin-fixed paraffin embedding (FFPE) after overnight fixation.

Fresh lung tumor tissue was obtained from an untreated 71-year-old lung cancer patient undergoing partial lung resection for cancer treatment at Kaplan Medical Center (Rehovot, Israel). After resection, the tissue was directly transported to the pathology department. A small sample of approximately 1 cm^3^ was taken and placed in ice-cold PBS for immediate transfer to the lab for ex-vivo organ culturing. All experiments were performed in accordance with Medical Ethics Committee approval and after obtaining informed consent. Specimen was coded anonymously prior to its arrival to the lab.

The human experiment was authorized by the Kaplan Medical Center (0097-18-KMC). IACUC authorization for the mice experiments was given by the IACUC committee of the Weizmann Institute.

### Data curation and normalization

Drug sensitivity measurements were downloaded from the Cancer Target Discovery and Development (CTD^2^) data portal (downloaded from https://ocg.cancer.gov/programs/ctd2/data-portal/). These data represented dose–response curve AUC measures for 887 cell lines across 481 compounds. Gene expression (Affymetrix HG-U133_Plus_2 Array Plate Set) and RNA-Seq of these cell lines was downloaded from the CCLE cell lines portal (https://portals.broadinstitute.org/ccle/data). The expression data were normalized by quantile normalization to produce RMA expression values from the Affymetrix CEL files^84^. RNA-sequencing data were downloaded as RPKM (Reads Per Kilobase of transcript per Million mapped reads) levels from the CCLE data portal.

The GDSC database is a resource for biomarker discovery for the development of therapeutics for cancer cells. It contains information from 138 anticancer drugs across 696 cell lines. These data were generated from high-throughput screening performed by the Cancer Genome Project at the Wellcome Trust Sanger Institute (WTSI) and the Center for Molecular Therapeutics at Massachusetts General Hospital. Gene-expression was quantified using an Affymetrix U219 mRNA expression array and was downloaded from the same portal. The expression data were normalized by quantile normalization to produce RMA expression values from the Affymetrix CEL files.

Project Achilles uses genome-scale RNAi and CRISPR-Cas9 genetic perturbation reagents to silence or knock out individual genes and identify those genes that affect cell survival. Gene essentiality profiles for 11,408 genes over 307 cell lines as well as 18,377 genes across 227 cell lines from genome-wide RNAi and CRISPR-Cas9 screens, respectively, were measured using the DEMETER algorithm, which aims to isolate the effects of gene knockdown from off-target effects. CERES is used to detect dependencies from the CRISPR data. Cell lines were designated as ‘sensitive’ or ‘not sensitive’ based on their Z-transformed RNAi and CRISPR essentiality levels.

Euclidean distance (ED) metric was applied to define the distance between the coordinates of any two genes or pathways in linear space. Hence, the smaller the distance between two genes or pathways, the more similar they are. Here, we calculated the similarity between the same platforms in different centers and between two different platforms by measuring the ED between the normalized gene expression or pathway-level activities across the cell lines in the corresponding datasets.

### Pathway network interactions dataset and analysis

Network information was obtained from the National Cancer Institute’s Pathway Interaction Database^26^, PharmGKB^27^, Wikipathways^28^, and SignaLinks^29^.

To calculate pathway activity metrics, we used the PathOlogist tool, which translates gene expression levels (in the manner detailed below) to a metric that provides information about the interactions within a pathway. This pathway activity is provided per sample, for each of the included pathways.

To use gene expression, PathOlogist first calculates, for each gene in each sample, the probability for that specific gene to be expressed in that specific sample. An expressed gene is called an “up” gene, and an unexpressed gene is called “down”. A probability for a specific gene to be in the “up” state is calculated using the distribution of the expression level of the gene across all samples.

To be able to accommodate a multitude of probability distributions, the algorithm uses gamma distributions as the family of functions that describes the gene expression. In the set of samples, the gene could be either expressed (“up”) or unexpressed (“down”) in each sample. Across samples, we assume that the collection of unexpressed instances follows an exponential distribution, which is one particular case of a gamma distribution. The collection of expressed instances is assumed to be distributed in a normal manner, which is well approximated by a larger mean gamma distribution. For each gene, we use an expectation-maximization (EM) algorithm to iterate over the data such that the likelihood of fitting these data by the distribution’s increases. The EM algorithm finally provides us with the most likely parameters of the modeled distributions and with the mixture weights of the two distributions. Once these two distributions are in place, we can calculate the probability of each gene in a sample to belong to one of the two distributions. That is, we obtain the probability of the gene to be in the up state. This probability is the (Up Down Probability) UDP measure that is used in the pathway score. Since a gene, in principle, could be in an “up” state or in a “down” state across all samples, we need to determine if the best fit is a single gamma distribution or a mixture of two gamma distributions. To compare these two models, we use the Bayesian information criterion (BIC).

Once we have the set of gene probabilities for each gene in each sample, we continue to calculate the pathway activity metric, which is calculated for 1028 pathways. PathOlogist treats the pathway as a network of interactions and assigns the network a score based on the expression levels of the interacting genes and on the quality of the interaction. The analysis also takes into consideration the specific type of interaction (inhibition or promotion). The activity of each interaction is calculated by multiplying the probability of the genes to be “active” (based on the UDP matrix). Then, the final pathway activity score is calculated by averaging all the interaction activity scores in the pathway. Once the pathway activity scores are calculated, a Mann–Whitney *U*-test is performed based on the sensitive/non-sensitive groups of cell lines in order to determine the dependency and significance of the association of the pathway activity scores with response to treatment.

### Gene set enrichment analysis (GSEA)

GSEA^40,41^ was applied to the microarray and RNA-seq data using the hallmark gene sets (*n* = 50) and the curated gene set (*n* = 4762) to discover statistically significant gene sets that can predict drug sensitivity.

### Gene set variation analysis (GSVA)

Similar to PathOlogist, GSVA^85^ calculates gene set enrichment scores per sample in an unsupervised manner (i.e., independent of any class label). GSVA was applied to both the microarray and RNA-seq data using the hallmark gene sets (*n* = 50) and the curated gene set (*n* = 4762). Mann–Whitney *U*-test was then applied on the GSVA results with the response groups, and FDR correction for multiple hypotheses was performed (as we did with the PathOlogist pathway activity scores). While MSigDB curated gene set (C2) did not yield any significant results, MSigDB Hallmark set yielded 654 significant results for the microarray dataset and 213 significant results for the RNA-seq dataset—none of which overlapped between the two sets.

### Sensitivity and power analysis

To test if the larger number of hypotheses generated by genes as compared to pathways was the reason that we did not detect any single gene as a predictive biomarker (Fig. 1), we re-ran the analysis with: (i) the 2512 genes that are part of the 1028 pathways and (ii) the 500 most variable genes. Consistent with our results using the full set of genes, no significant or near-significant individual genes were found in the set of 2512 genes in the pathways. Only one near-significant hit was found in the top 500 most variable genes (*SPP1*, high expression levels were associated with sensitivity to GDC-0879, a *BRAF* inhibitor, in skin cancer cell lines; *q* = 0.228), and this gene did not belong to any of the significant pathways.

To test whether and how the number of cell lines affected the number of significant results, we performed ‘down-sampling’ analysis on the NSCLC (*n* = 106) and skin (*n* = 50) subsets of cell lines. Specifically, we explored how the number of significant results decreased with sample size by repeating the analysis on smaller random subsets of the cell lines, reducing the subset to as few as 10 cell lines per tumor type. As expected, for both sets of cell lines, the number of significant pathways (*q* < 0.25) increased with the number of cell lines analyzed (Supplementary Fig. 4a, b), suggesting the importance of analyzing a large number of cell lines per tumor type (at least 30) in order to discover significant tumor-specific pathways.

### RNA extraction and quantitative real-time polymerase chain reaction (qRT-PCR)

Total RNA was purified using a Qiagen RNeasy mini-prep kit (product #74104) according to the manufacturer’s protocol. A 0.5-μg aliquot of total RNA from each sample was reverse transcribed using PCRBIO cDNA Synthesis kit (product #PB30.11-10). qRT-PCR was performed on the Applied Biosystems qPCR system using KAPA SYBR Green Fast ABI Prism qPCR kit (Kapa Biosystem, product #KK4605) at 94 °C for 3 min to denature RNA, with 40 cycles of amplification at 94 °C for 15 s, 50 °C for 30 s, and 72 °C for 30 s; Data analysis was performed according to the ΔΔCt Method by normalizing the expression level of each gene to that of a glyceraldehyde 3-phosphate de-hydrogenase (*GAPDH*) reference gene in the same sample.

The following primers were used for qRT-PCR: *AIFM:* 5′-AAGGGCAATGCAGACTACAGA (F) and 5′-GGAACCATCATGTGCCCAAAG (R); *BCL2L1:* 5′-ATTGGTGAGTCGGATCGCAG (F) and 5′-CCACAAAAGTATCCCAGCCG (R); *PARP1:* 5′-CGAGTCGAGTACGCCAAGAG (F) and 5′-CATCAAACATGGGCGACTGC (R); and *GAPDH:* 5′-ACCCACTCCTCCACCTTTG (F) and 5′-CTCTTGTGCTCTTGCTGGG (R).

### Drug screen

On day 0, GFP-labeled cancer cells (6000 cells/well in 120 μL) were plated in 96-well clear-bottom plates (Greiner, product #60-655090). On day 1, the cells were treated with 15 μL 10× of drug A and 15 μL 10× of drug B or DMSO (Sigma-Aldrich Cat #D2650-100ML; See Supplementary Table 1) using the CyBi-Well Vario 96/250 Simultaneous Pipette (CyBio). On day 4, the medium in all wells was replaced with a fresh medium containing the same drugs that were applied on day 1. GFP fluorescence was read on days 1, 4, and 7 using the Cytation 3 cell-imaging Multi-Mode reader (BioTek). Screens were carried out in duplicate. ABT-263 (Cat #A10022) and Vinorelbine (Cat #A10976) were purchased from AdooQ Bioscience.

### Tissue immunohistochemistry

Immunohistochemistry was performed on 4-μm sections from FFPE tissue samples from ex-vivo organ culture. Hematoxylin and Eosin (H&E) staining was performed using an automated stainer. Cleaved Caspase-3 staining (Cell Signaling Antibody (Asp175); 1:1000 dilution) was performed using an automated stainer (BOND RX, Leica Biosystems, Rhenium, Modiin, Israel).

### Tissue viability and scoring

Human tissue was scored using pathological criteria of response to treatment commonly used after neoadjuvant therapy as recommended by the College of American Pathologists (Modified Ryan Scheme for Tumor Regression Score). A score of 0 represents no viable cancer cells, and a score of 3 represents extensive residual cancer with no evident regression (poor or no response).

### Statistical analysis

Dose–response AUCs (dr-AUC) for 481 compounds from the CTD^2^ dataset were calculated by fitting a curve through viability readouts across 16 different concentrations of a compound in a given cell line. A dr-AUC value of 0 represents complete cell death at all drug concentrations, while a dr-AUC value of 15 represents a “flat” curve (with 100% viability [i.e., no killing] across all concentrations). dr-AUC values greater than 15 indicate an increasing curve (viability >100% at high concentrations, indicating potential increased cell proliferation due to the drug). The dr-AUC values were further centered and normalized to yield *Z*-scores for every compound across all cell lines. The *Z*-scores were then used to define relatively sensitive and non-sensitive cell lines: cell lines with a *Z*-score < −1.5 were designated as sensitive to the particular compound, and cell lines with a *Z*-score > 0 were tagged as non-sensitive. Cell lines with a Z-score between −1.5 and 0 were excluded from further analysis in order to avoid contaminating the sensitive and non-sensitive groups with intermediate cases and thus obscure the signal. Eighteen cell lines that were sensitive to more than 20% of the compounds were excluded from the analysis. In addition, pathways that were not variable across the cell lines were excluded from further analysis (219 pathways calculated from the microarray data and 217 from the RNA-Seq data; 181 overlapped). We removed pathways with a range of activity scores ≤ 0.1 across samples (note that the activity scores can range between 0 and 1).

The data were partitioned according to cancer type (overall 10 cancer types), and a two-sided Mann–Whitney *U*-test was used to identify pathways whose activity level was significantly associated with the sensitivity of a specific cancer type to a specific compound or essentiality of a specific gene (obtained by RNAi/CRISPR-Cas9). In every tissue type, compounds with minimal AUC level greater than 8 were omitted from further analysis. This was done in order to only include compounds with actual response.

For each tissue type, we calculated the significance (i.e., *P* value) for every pair of pathway and compound (overall ~2 × 10^6^ pathway, compound, and tissue-type combinations), and for every pair of pathway and RNAi/CRISPR-Cas9 gene (overall ~46 × 10^6^ possible combinations). We then corrected for multiple hypothesis testing all *P* values per tissue type using the Benjamini–Hochberg FDR procedure^42^ . Results with FDR *q* < 0.05 were considered as significant, and those with 0.05 < *q* < 0.25 were tagged as near-significant.

We produced a quantile–quantile (QQ) plot (Fig. 1e and Supplementary Fig. 1k–m) of *P* value quantiles to compare the observed distribution of all *P* values per tissue type and platform with the expected *P* values under the null hypothesis. Dots (results) that lie on the *y* = *x* line follows the null hypothesis, while dots that lie above the diagonal represent significant results.

### Down-sampling analyses

To analyze the dependency between the number of significant results and the number of cell lines in the dataset, we performed down-sampling analysis on NSCLC, and skin cell lines, separately. We repeated our analysis pipeline by randomly subsampling the data using different numbers of cell lines, beginning at a minimum of 10 cell lines. Subset sizes were chosen on the basis of sampling at ascending order from 10 cell lines to the final number of cell lines in the given tissue type. For each of the random subsets, we repeated the full analysis 10 times.

### Dimensionality reduction

The t-Distributed Stochastic Neighbor Embedding (t-SNE) method^33^ was used for dimensionality reduction with the default perplexity parameter of 30. Of note, t-SNE was used only for visualization and not for clustering.

### Cluster robustness analysis

To assess the robustness of the clusters, we used the following three internal clustering validity indices. (i) The Calinski–Harabasz^35^ index, which calculates the proportion between the dissimilarity (or the distance) between clusters and tightness or the dissimilarity within the cluster. (ii) The Dunn index^36^ aims to identify sets of clusters that are compact with a small variance between the members and are well separated. (iii) Silhouette scores^34^ calculate, on average, how members within a cluster are closely grouped and, at the same time, how loosely these members belong to neighboring clusters.

### Reporting summary

Further information on research design is available in the Nature Research Reporting Summary linked to this article.

## Supplementary information

Supplementary Information

Description of Additional Supplementary Files

Supplementary Data 1

Supplementary Data 2

Supplementary Code 1

Reporting Summary

## Data Availability

The CTD^2^ dataset is available in the CTD^2^ data portal under the following link (https://ocg.cancer.gov/programs/ctd2/data-portal). The AUC levels and cell lines that were analyzed in this paper are listed in Supplementary Data 1. Cell line RNA-seq and microarray expression was downloaded from the CCLE portal (the same cell lines from Supplementary Data 1). RNAi and CRISPR-Cas9 screens were downloaded from the Achilles project data portal https://depmap.org/portal/achilles/. The different datasets that were used for validation experiments are publicly available as follows: (a) The Yang et al. drug screen and expression data was downloaded from the GDSC data portal https://www.cancerrxgene.org/; (b) human lung, thyroid, and breast tumors from TCGA were downloaded from the GDC data portal https://portal.gdc.cancer.gov/; (c) Project DRIVE validation sets were downloaded from the DRIVE data portal https://oncologynibr.shinyapps.io/drive/; (d) Rizos et al. dataset is available in GEO under the accession number: GSE50509; (e) Tse et al. dataset is available in GEO under the accession number: GSE10841; (f) Girard et al. dataset is available in GEO under the accession number: GSE31547; (g) Kadara et al. dataset is available in GEO under the accession number: GSE44077; (h) Spira et al. dataset is available in GEO under the accession number: GSE4115; (i) Pilar et al. dataset is available in GEO under the accession number: GSE70541; (j) Piccolo et al. dataset is available in GEO under the accession number: GSE47862; (k) Jönsson et al. dataset is available in GEO under the accession number: GSE25307; and (l) Lisowska et al. dataset is available in GEO under the accession number: GSE50567.

## Code availability

The code and datasets are available in GitHub at the following link: https://github.com/getzlab/pathway_based_biomarker.
