# Supplementary material for: Predicting and affecting response to cancer therapy based on pathway-level biomarkers
Source: Nat Commun. 2020 Jul 3;11:3296. doi: 10.1038/s41467-020-17090-y (PMC7335104; doi:10.1038/s41467-020-17090-y)
Supplement: Supplementary file 1 — Supplementary Information [file 41467_2020_17090_MOESM1_ESM.pdf]

Supplementary Figure 1

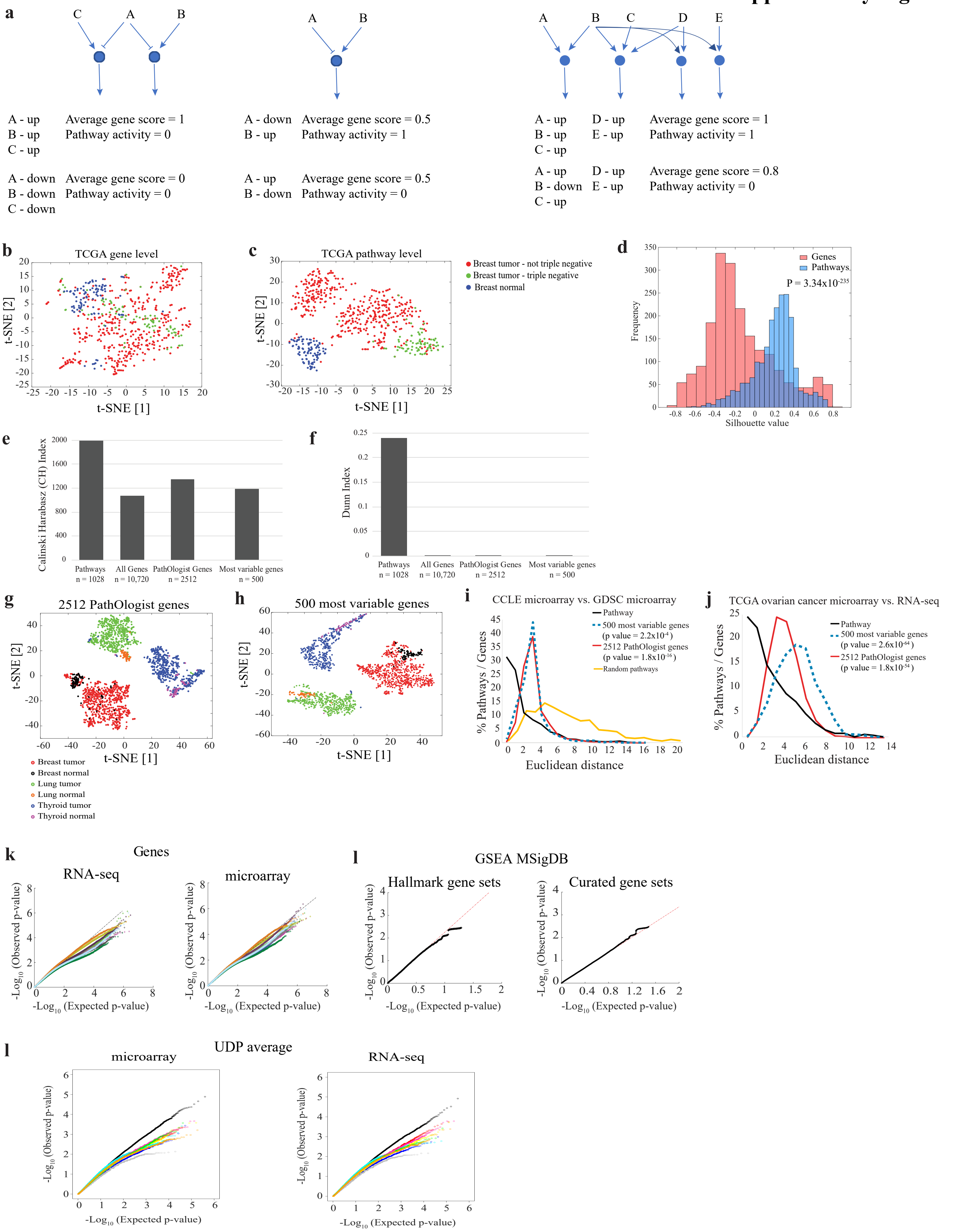

Supplementary Figure 1. Pathways form more robust clusters related to Figure 1

(A) A gene/protein participates in multiple interactions. That gene could enhance the activity of one interaction while reducing the activity of another interaction. This could not be captured by gene membership. See Figure "1"

The co-dependency of an interaction on multiple genes leads to activity values that could, for example, enhance with the heightened co-expression of the gene, or alternatively reduce with the co-expression of the genes. This behavior is dependent on the specific form of co-expression of the genes and could not be captured by gene membership. See Figure "2"

Relative weight of the influence of specific genes over entire pathway activity scores relies heavily on the role of specific genes within the pathway. A hub protein/gene might influence greatly over the pathway, but this influence might be completely irrelevant to the pathway score if, for example, that gene is involved in interactions with other specific genes that are unexpressed. The intricacy of the pathway narrative does not translate at all to gene membership. See Figure "3"

(B) tSNE plot of the gene-expression levels in TCGA breast cancer patients. Samples are colored by tissue subtype and state: (i) non–triple-negative breast cancer in red, (ii) triple-negative breast cancer in green, and (iii) normal adjacent breast tissue.

(C) tSNE plot of the pathway levels in TCGA breast cancer patients. Samples are colored by tissue subtype and state.

(D) Gene and pathway cluster robustness analysis was evaluated using three indices in (C), (D), and (E). Here, silhouette value distribution is compared in genes and pathways.

(E) Calinski-Harabasz index.

(F) Dunn index. High values represent more compact clusters.

(G) tSNE plot of the 2512 PathOlogist genes in three tumor types and their adjacent normal tissue. Samples are colored by tissue type and state (tumor/normal).

(H) tSNE plot of the 500 most variable genes in three tumor types and their adjacent normal tissue.

(I) ED distribution of 2512 PathOlogist genes and pathways calculated from microArray from two different institutions (CCLE and GDSC) across the same 438 cell lines. Blue line represents the ED distribution between the pathways, and the red line represents the ED distribution between the genes (left panel: 2512 PathOlogist genes; right panel: 500 most variable genes).

(J) ED distribution of genes and pathways between RNA-seq and microArray across 294 ovarian cancer patients. Blue line represents the ED distribution between the pathways, and the red line represents the ED distribution between the genes (left panel: 2512 PathOlogist genes; right panel: 500 most variable genes).

(K) qq-plot of the single gene results

(L) qq-plot of GSEA results (hallmark and C2)

(M) qq-plot of UDP average

Supplementary Figure 2

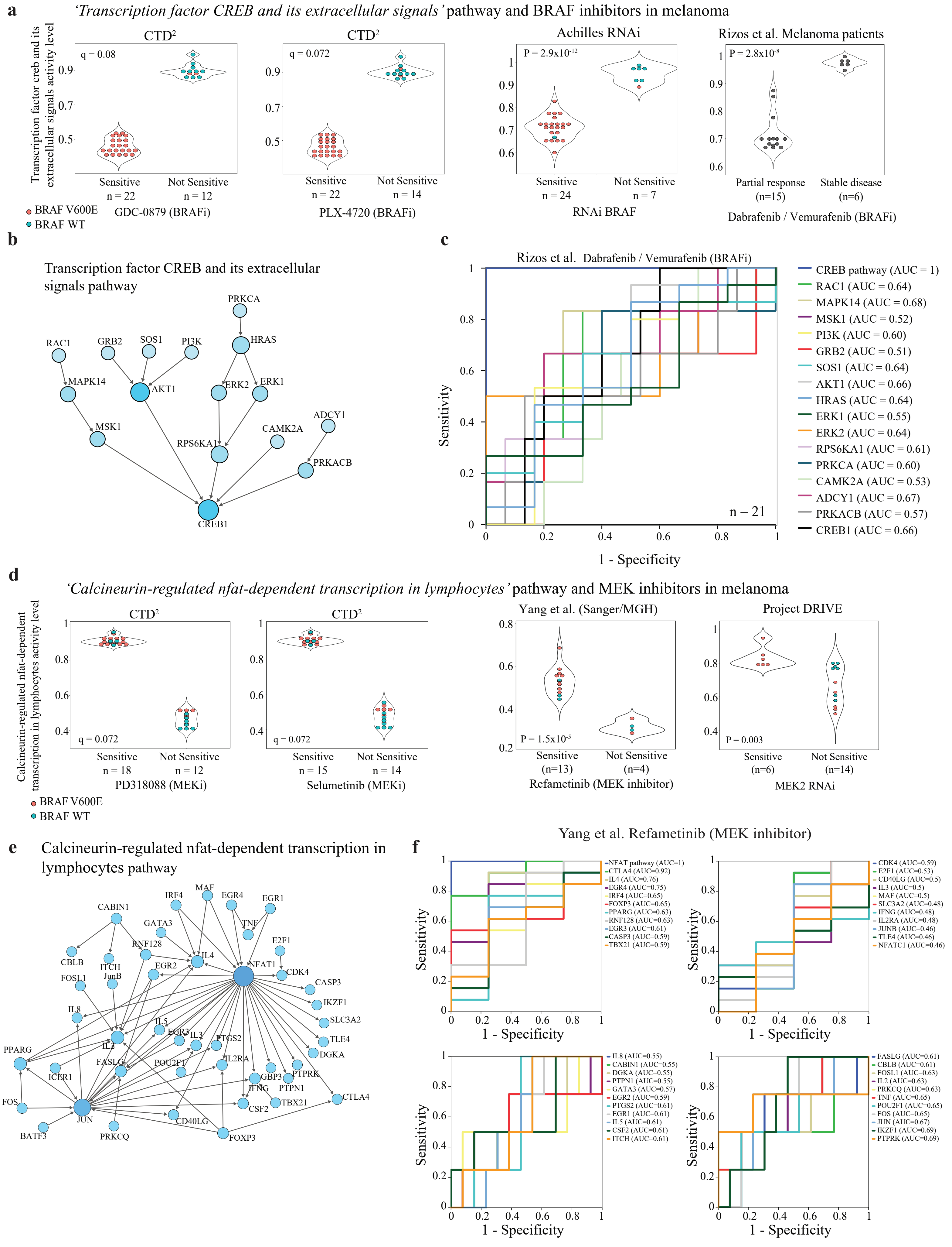

Supplementary Figure 2. Positive control validation and down-sampling analysis related to Figure 1

(A) Violin plots of *'Transcription factor CREB and its extracellular signals'* pathway and BRAF inhibitor results from the CTD^2 and validation from two independent cohorts. Dots are colored by BRAF V600E mutation status (pink represents mutated BRAF and blue represents wild type).

(B) Network diagram representing the '*Transcription factor CREB and its extracellular signals'* pathway.

(C) Receiver operating characteristic (ROC) analysis was constructed to evaluate the prognostic power of the *CREB* pathway versus all the genes in this pathway. The area under the ROC curve (AUC) was used to quantify response prediction.

(D) Violin plot of '*Calcineurin-regulated nfat-dependent transcription in lymphocytes'* pathway and MEK inhibitor results from CTD^2 and validation from two independent cohorts. Dots are colored by BRAF V600E mutation status (pink: mutated BRAF; blue: wild type).

(E) Network diagram representing the '*Calcineurin-regulated nfat-dependent transcription in lymphocytes'* pathway.

(F) ROC analysis was constructed to evaluate the prognostic power of the *NFAT* pathway versus all the genes in this pathway. The AUC was used to quantify response prediction.

Supplementary Figure 3

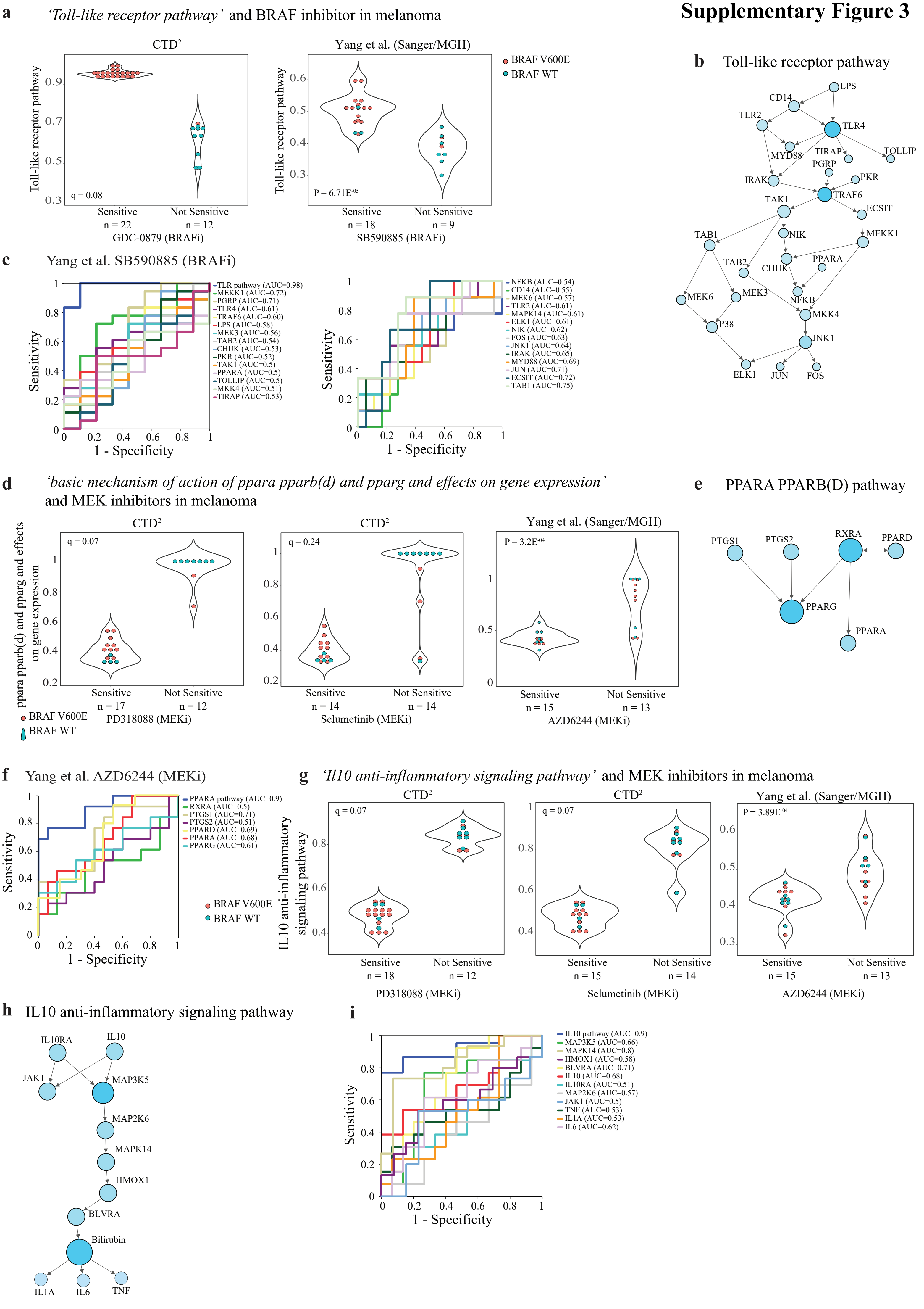

Supplementary Figure 3. Results validation related to Figure 1

(A) Violin plot of '*Toll-like receptor pathway'* and BRAF inhibitor results from CTD^2 and validation from the GDSC Yang et al. cohort. Dots are colored by BRAF V600E mutation status (pink: mutated BRAF; blue: wild type).
(B) Network diagram representing the '*Toll-like receptor pathway'*.
(C) ROC analysis was constructed to evaluate the prognostic power of the *'Toll-like receptor pathway'* versus the pathway genes in the validation set. The AUC was used to quantify response prediction.
(D) Violin plot of '*basic mechanism of action of ppara pparb(d) and pparg'* pathway and MEK inhibitor results from CTD^2 and validation from the GDSC Yang et al. cohort. Dots are colored by BRAF V600E mutation status.
(E) Network diagram representing the '*basic mechanism of action of ppara pparb(d) and pparg'.*
(F) ROC analysis was constructed to evaluate the prognostic power of the '*basic mechanism of action of ppara pparb(d) and pparg'* versus the pathway genes in the validation set.
(G) Violin plot of '*IL10 anti-inflammatory signaling pathway'* and MEK inhibitor results from CTD^2 and validation from the GDSC Yang et al. cohort. Dots are colored by BRAF V600E mutation status.
(H) Network diagram representing the '*IL10 anti-inflammatory signaling pathway'.*
(I) ROC analysis was constructed to evaluate the prognostic power of the '*IL10 anti-inflammatory signaling pathway'* versus the pathway genes in the validation set.

Supplementary Figure 4

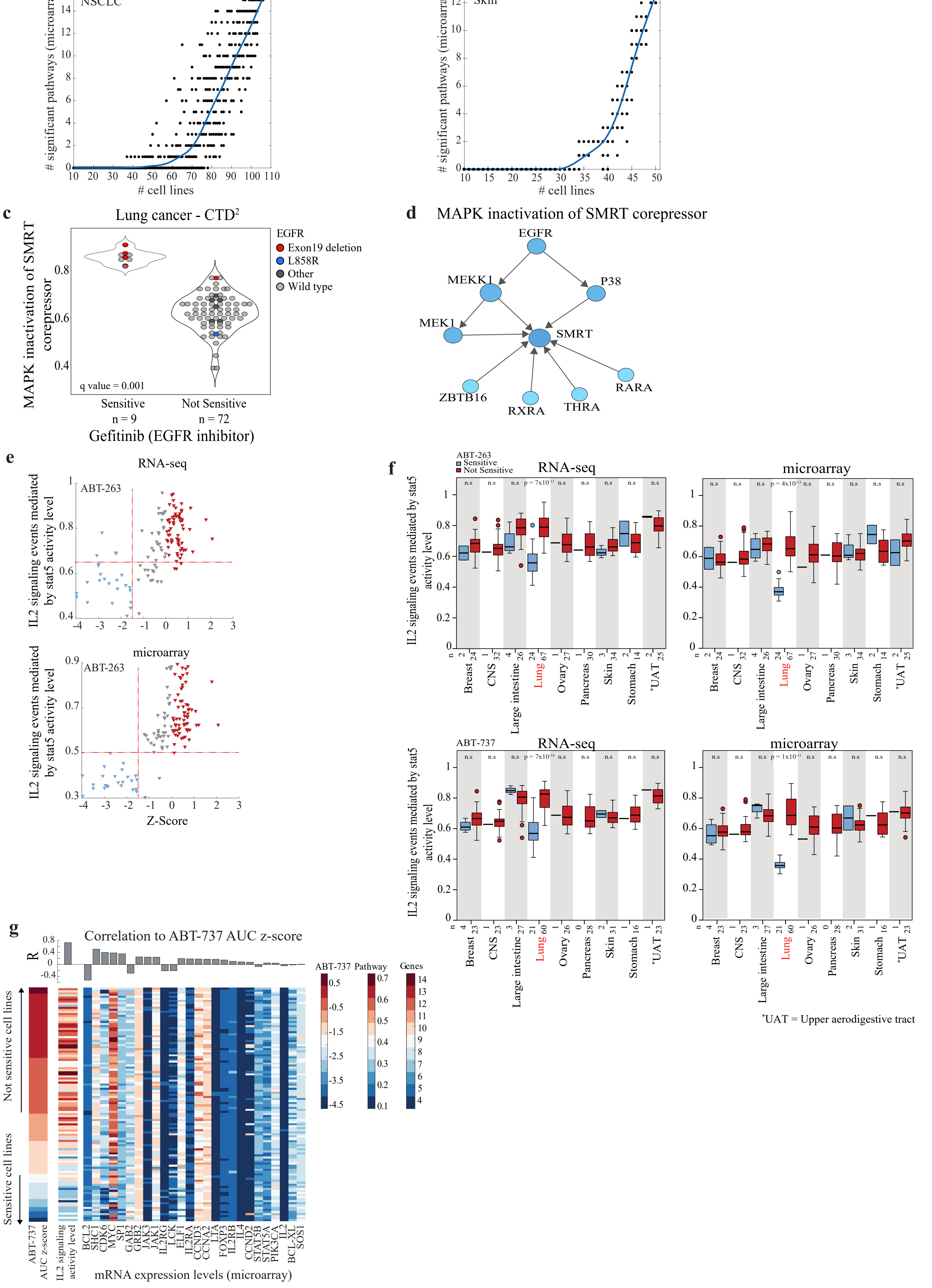

Supplementary Figure 4.

(A) Down-sampling analysis within NSCLC cell lines shows that significant pathway biomarker discovery continues to accumulate with increasing number of samples. Each point represents a random subsampling of the cell lines starting from 10 cells; every iteration was repeated 10 times. X-axis indicates the number of cell lines in the sample; Y-axis indicates the number of significant pathways. Blue line is a smoothed fit.

(B) Down-sampling analysis within skin cell lines.

(C) 'MAPK inactivation of SMRT corepressor' pathway and Gefitinib (EGFR inhibitor) as a predictor of EGFR exon 19 deletion.

(D) Network diagram representing the '*MAPK inactivation of SMRT corepressor*' pathway.

(E) Scatter plot of ABT-263 AUC z-score values versus IL2–STAT5 pathway levels in microArray and RNA-seq (Blue: sensitive cell lines; Red: not-sensitive cell lines; Gray: samples that were excluded from the analysis).

(F) IL2–STAT5 pathway activity levels in sensitive and not-sensitive cell lines in microArray and RNA-seq across all tissue types.

(G) HeatMap of all genes in the IL2-STAT5 signaling pathway

Supplementary Figure 5

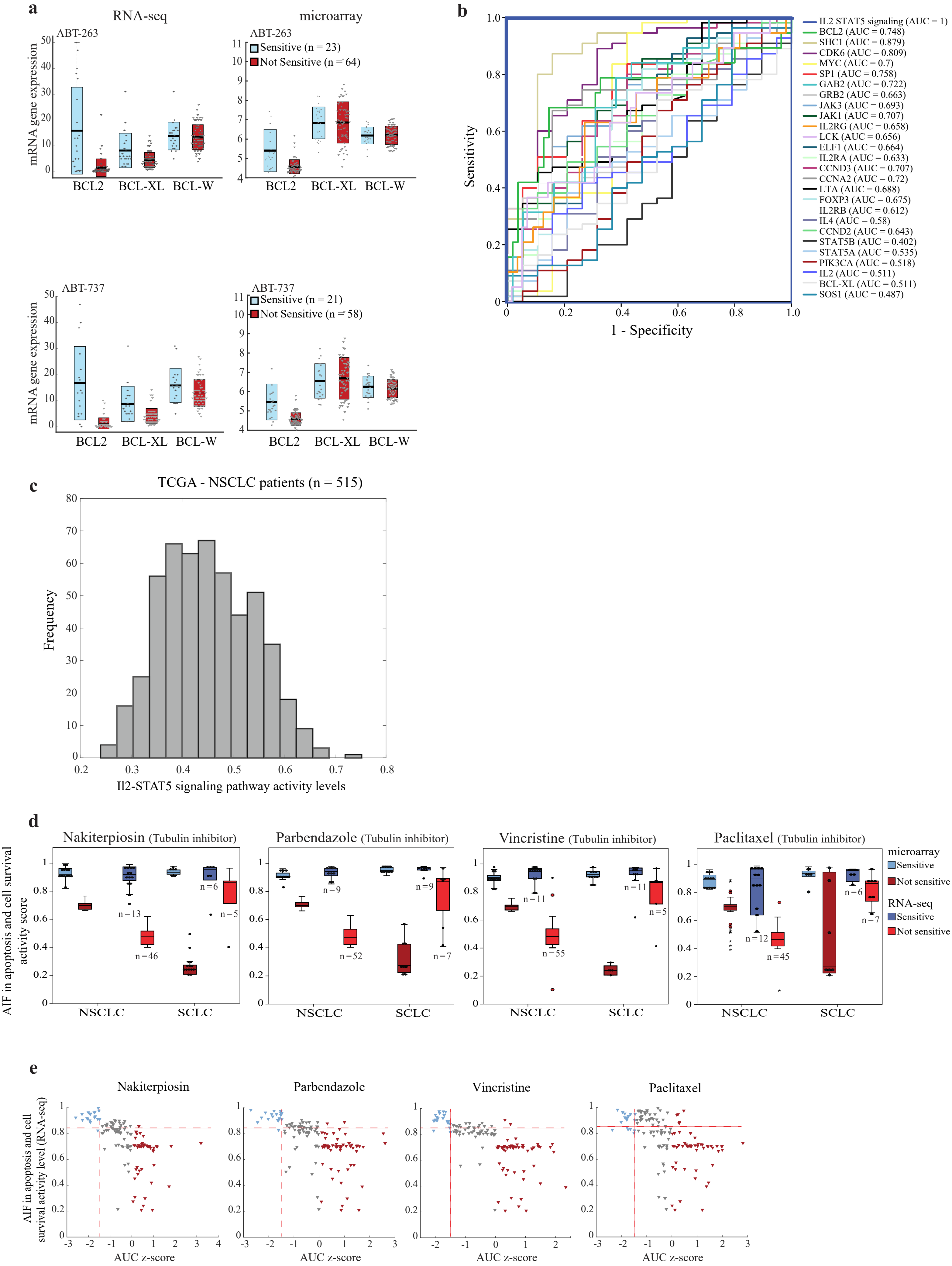

Supplementary Figure 5.

(A) Box plots represent *BCL2, BCL-XL*, and *BCL-W* expression levels in sensitive and not-sensitive lung cancer cell lines to MTI’s. Error bars represent the standard deviation.

(B) ROC analysis was constructed to evaluate the prognostic power of the ‘*IL2-STAT5 signaling pathway’* versus the pathway genes in the validation set.

(C) IL2–STAT5 pathway activity levels distribution in TCGA lung adenocarcinoma patients.

(D) *‘AIF in Apoptosis and cell-survival pathway’* activity levels in sensitive (blue) and not-sensitive (red) cell lines across 9 tissue types in microarray and RNA-seq data. Cell lines in red indicate lung cancer cell lines. Error bars represent the standard deviation.

(E) Scatter plot of the 4 MTI’s AUC z-score values versus AIF pathway activity levels in RNA-seq data. Blue triangles represent sensitive cell lines, and red triangles represent not-sensitive cell lines (gray triangles represent samples that were excluded from the analysis).

Supplementary Figure 6

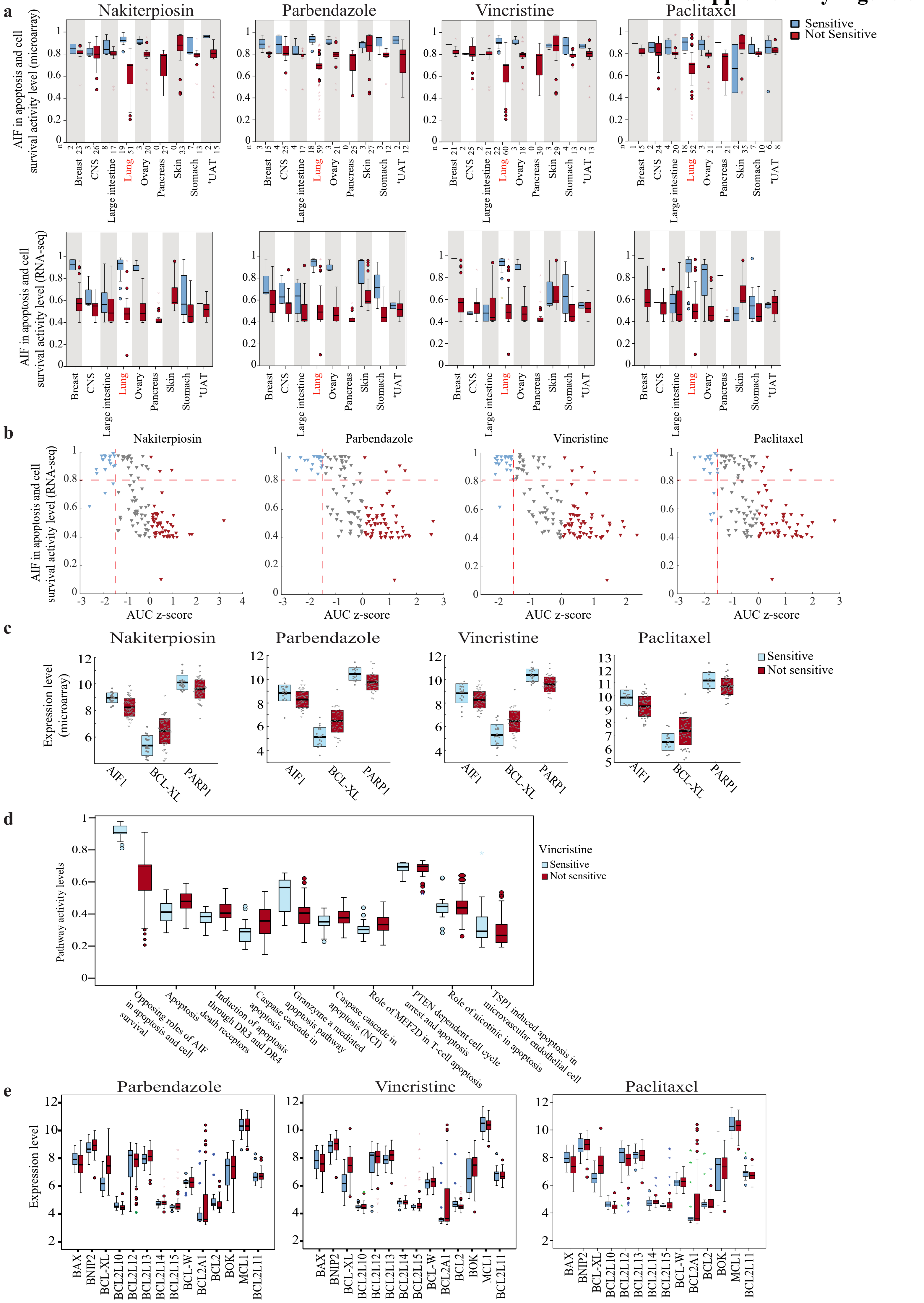

Supplementary Figure 6. ***AIF in apoptosis and cell survival pathway***

(A) *'AIF in Apoptosis and cell-survival pathway'* activity levels in sensitive and not-sensitive cell lines across 9 tissue types in microArray and RNA-seq data. Error bars represent the standard deviation.

(B) Scatter plot of the 4 MTI's AUC z-score values versus AIF pathway activity levels in RNA-seq data. Blue triangles represent sensitive cell lines, and red triangles represent not-sensitive cell lines (gray triangles represent samples that were excluded from the analysis).

(C) Box plots represent *AIF1, BCL-XL*, and *PARP1* expression levels in sensitive and not-sensitive lung cancer cell lines to MTI's. Error bars represent the standard deviation.

(D) Box plots represent all apoptosis-related pathway activity levels in sensitive and not-sensitive lung cancer cell lines to vincristine. Error bars represent the standard deviation.

(E) Box plot of BCL2 protein–family member expression levels in sensitive (blue boxes) and not-sensitive (red boxes) cell lines to the MTI's parbendazole, vincristine, and paclitaxel. Error bars represent the standard deviation.

Supplementary Figure. 7

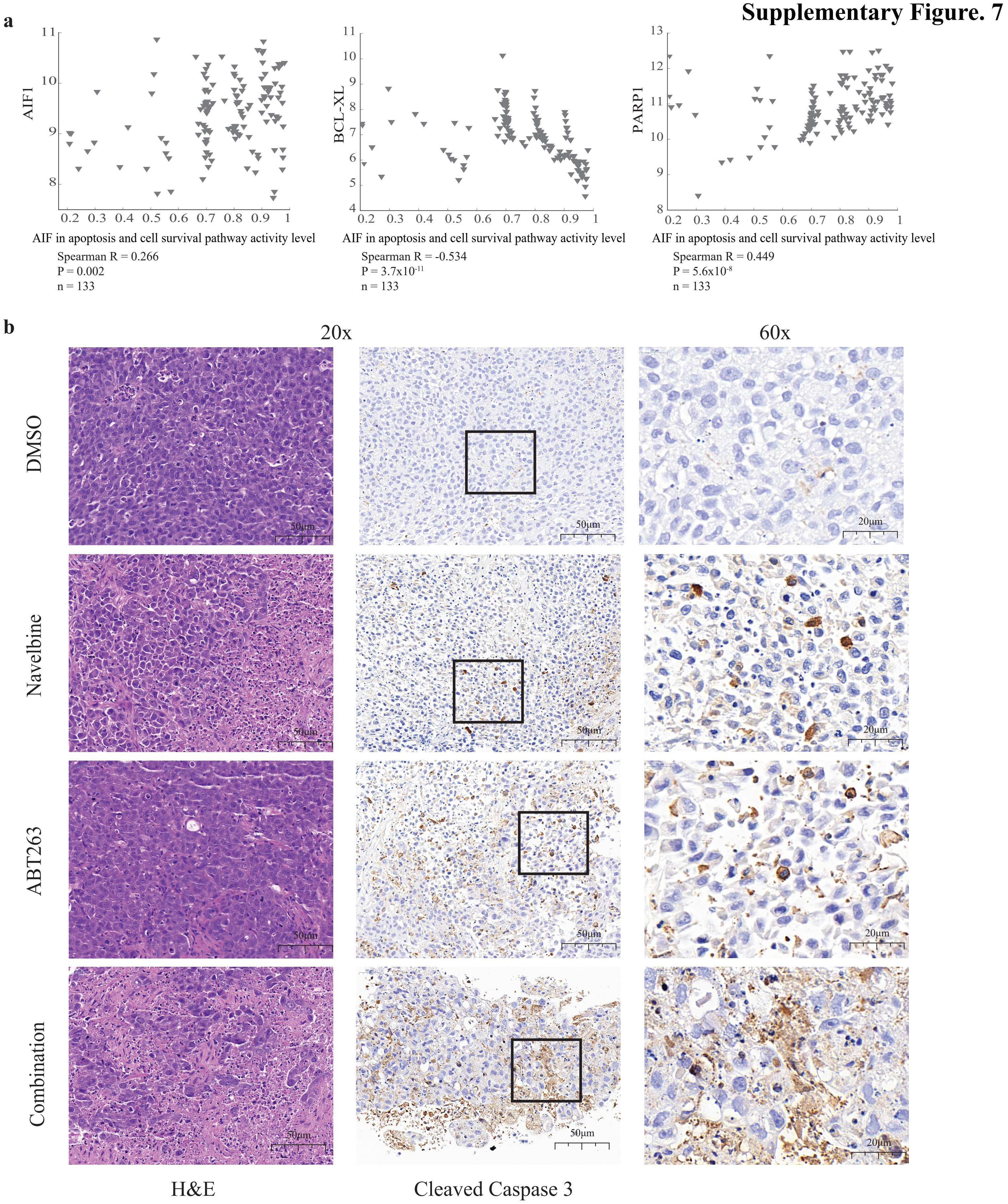

Supplemetary Figure 7. FFPEs cleaved caspase-3 staining

(A) Scatter plot of 'AIF in Apoptosis and cell-survival pathway' activity levels versus the mRNA expression levels of AIF1, BCL-XL, and PARP1 in lung cancer cell lines. (B) H&E and cleaved caspase-3 staining of NSCLC PDX tumors treated ex-vivo with DMSO (control), Navelbine alone, ABT-263 alone, and a combination of the two drugs.

Supplementary Figure 8

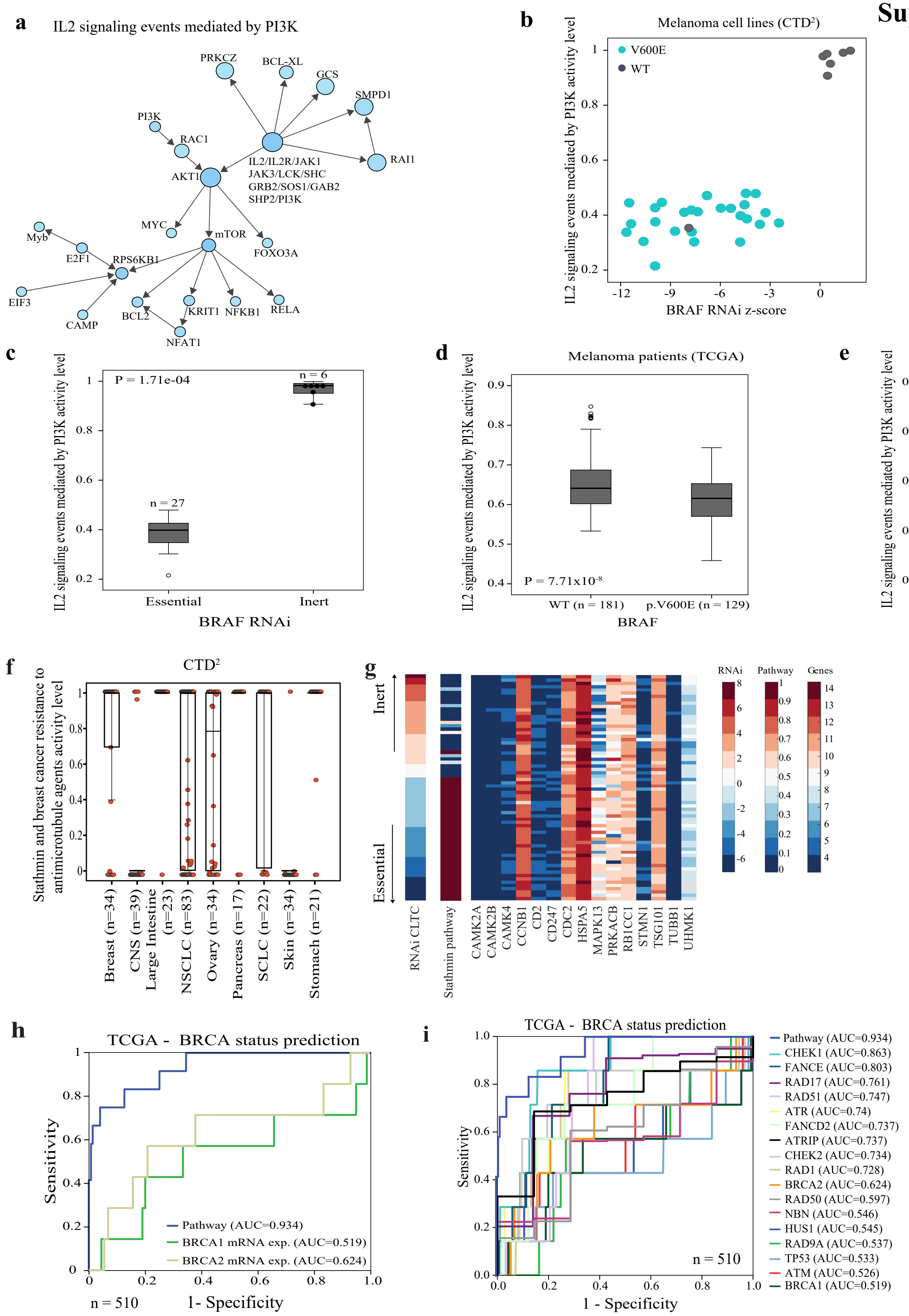

Supplementary Figure 8. *IL2 signaling events mediated by PI3K*

(A) Network diagram representing the *'IL2 signaling events mediated by PI3K'* pathway.

(B) Scatter plot of BRAF RNAi z-score values versus *'IL2 signaling events mediated by PI3K'* pathway activity levels in melanoma cell lines. Circles are colored by the presence of the BRAF V600E mutation (blue: *BRAF* V600E mutation; gray: wild-type).

(C) Box plot represents *'IL2 signaling events mediated by PI3K'* pathway activity levels in CTD^2 melanoma cell lines with the *BRAF* V600E mutation or wild-type *BRAF*. Error bars represent the standard deviation.

(D) Box plot represents *'IL2 signaling events mediated by PI3K'* pathway activity levels in TCGA melanoma patients with the *BRAF* V600E mutation or wild-type *BRAF*. Error bars represent the standard deviation.

(E) Box plot represents *'IL2 signaling events mediated by PI3K'* pathway activity levels in melanoma patients (Barras et al.) with the *BRAF* V600E mutation or wild-type *BRAF*. Error bars represent the standard deviation.

(F) Stathmin pathway activity level distribution in 9 tissue types. Every dot represents a cell line. Error bars represent the standard deviation.

(G) Heatmap of Stathmin pathway 13 genes, *CLTC*, and Stathmin pathway activity levels versus CLTC RNAi essentiality score, sorted by essentiality.

(H) ROC analysis was constructed to evaluate the prognostic power of the BRCA pathway activity levels versus the expression levels of *BRCA1* and *BRCA2* in TCGA breast cancer dataset. The AUC was used to quantify response prediction.

(I) ROC of the BRCA pathway and the 17 pathway genes in the TCGA breast cancer dataset.

Supplementary Table 1 – Drugs:

	Supplier	Catalog number
DMSO	Sigma Aldrich	D2650-100ML
ABT-263	AdooQ Bioscience	A10022
Vinorelbine	AdooQ Bioscience	A10976
