# Supplementary material for: Predicting and affecting response to cancer therapy based on pathway-level biomarkers
Source: Nat Commun. 2020 Jul 3;11:3296. doi: 10.1038/s41467-020-17090-y (PMC7335104; doi:10.1038/s41467-020-17090-y)
Supplement: Supplementary file 2 — Description of Additional Supplementary Files [file 41467_2020_17090_MOESM2_ESM.pdf]

Description of Additional Supplementary Files

File Name: Supplementary Data 1
Description: CTD2 AUC levels of all compounds that were analyzed in this work across all cell lines.

File Name: Supplementary Data 2
Description: PathOlogist pathway activity levels across all cell lines.

File Name: Supplementary Code 1
Description: This folder contains the following files:

1) Drug_Pw_RankSum_Tissue_Specific.m - The main function that recieves as input pathway activity levels or gene expression and drug response AUCs

2) Data.xlsx - This is the input data that was used in this paper (CTD2 pathway activity levels, gene expression and AUCs Z-score)

3) Print_significant_results.m - a sub-function used in the main code(1) for printing results with FDR < 0.25.

4) QQ_PLOT.m - calculation of qq plot for evaluation of the results per tissue type.

5) Down_sampling.m - down samplinh analysis that was performed as disscused in the MS.

The PathOlogist is an open source tool that was developed in the NIH and can be access via the following paper: - PMID: 21542931.
