# Supplementary material for: Predicting and affecting response to cancer therapy based on pathway-level biomarkers
Source: Nat Commun. 2020 Jul 3;11:3296. doi: 10.1038/s41467-020-17090-y (PMC7335104; doi:10.1038/s41467-020-17090-y)
Supplement: Supplementary file 6 — Reporting Summary [file 41467_2020_17090_MOESM6_ESM.pdf]

nature research

Corresponding author(s): Gad Getz and Ravid Straussman

Last updated by author(s): June 1st, 2020

Reporting Summary

Nature Research wishes to improve the reproducibility of the work that we publish. This form provides structure for consistency and transparency in reporting. For further information on Nature Research policies, see Authors & Referees and the Editorial Policy Checklist .

Statistics

For all statistical analyses, confirm that the following items are present in the figure legend, table legend, main text, or Methods section.

n/a	Confirmed	
☐	☒	The exact sample size (*n*) for each experimental group/condition, given as a discrete number and unit of measurement
☐	☒	A statement on whether measurements were taken from distinct samples or whether the same sample was measured repeatedly
☐	☒	The statistical test(s) used AND whether they are one- or two-sided<br>*Only common tests should be described solely by name; describe more complex techniques in the Methods section.*
☒	☐	A description of all covariates tested
☐	☒	A description of any assumptions or corrections, such as tests of normality and adjustment for multiple comparisons
☐	☒	A full description of the statistical parameters including central tendency (e.g. means) or other basic estimates (e.g. regression coefficient) AND variation (e.g. standard deviation) or associated estimates of uncertainty (e.g. confidence intervals)
☐	☒	For null hypothesis testing, the test statistic (e.g. *F*, *t*, *r*) with confidence intervals, effect sizes, degrees of freedom and *P* value noted<br>*Give P values as exact values whenever suitable.*
☒	☐	For Bayesian analysis, information on the choice of priors and Markov chain Monte Carlo settings
☒	☐	For hierarchical and complex designs, identification of the appropriate level for tests and full reporting of outcomes
☐	☒	Estimates of effect sizes (e.g. Cohen's *d*, Pearson's *r*), indicating how they were calculated

Our web collection on statistics for biologists contains articles on many of the points above.

Software and code

Policy information about availability of computer code

Data collection	All data used was downloaded from publicly available sources (GEO, TCGA, CTD2, Sanger/MGH, Achilles project, Project DRIVE)
Data analysis	All the code in this study was written in Matlab 2017b.

For manuscripts utilizing custom algorithms or software that are central to the research but not yet described in published literature, software must be made available to editors/reviewers. We strongly encourage code deposition in a community repository (e.g. GitHub). See the Nature Research guidelines for submitting code & software for further information.

Data

Policy information about availability of data

All manuscripts must include a data availability statement. This statement should provide the following information, where applicable:

- Accession codes, unique identifiers, or web links for publicly available datasets
- A list of figures that have associated raw data
- A description of any restrictions on data availability

The CTD2 dataset is available in the CTD2 data portal under the following link (https://ocg.cancer.gov/programs/ctd2/data-portal). The AUC levels and cell lines that were analyzed in this paper are listed in Supplementary Table 1. Cell line RNA-seq and microarray expression was downloaded from the CCLE portal (the same cell lines from Supplementary Table 1). RNAi and CRISPR-Cas9 screens were downloaded from the Achilles project data portal https://depmap.org/portal/achilles/.
The different datasets that were used for validation experiments are publicly available as follows: (a) The Yang et al. drug screen and expression data was downloaded from the GDSC data portal https://www.cancerrxgene.org/; (b) Human lung, thyroid, and breast tumors from TCGA were downloaded from the GDC data portal https://portal.gdc.cancer.gov/; (c) Project DRIVE validation sets were downloaded from the DRIVE data portal https://oncologynibr.shinyapps.io/drive/; (d) Rizos et al. dataset is available in GEO under the accession number: GSE50509 https://www.ncbi.nlm.nih.gov/geo/query/acc.cgi?acc=GSE50509; (e) Tse et al. dataset is available in GEO under the accession number: GSE10841 https://www.ncbi.nlm.nih.gov/geo/query/acc.cgi?acc=GSE10841; (f) Girard et al. dataset is available in GEO under the accession number: GSE31547 https://www.ncbi.nlm.nih.gov/geo/query/acc.cgi?acc=GSE31547; ; (g) Kadara et al. dataset is available in GEO under the accession number: GSE44077 https://www.ncbi.nlm.nih.gov/geo/query/acc.cgi?acc=GSE44077; (h) Spira et al. dataset is available in GEO under the

accession number: GSE4115 https://www.ncbi.nlm.nih.gov/geo/query/acc.cgi?acc=GSE4115; (i) Pilar et al. dataset is available in GEO under the accession number: GSE70541 https://www.ncbi.nlm.nih.gov/geo/query/acc.cgi?acc=GSE70541; (j) Piccolo et al. dataset is available in GEO under the accession number: GSE47862 https://www.ncbi.nlm.nih.gov/geo/query/acc.cgi?acc=GSE47862; (k) Jonsson et al. dataset is available in GEO under the accession number: GSE25307 https://www.ncbi.nlm.nih.gov/geo/query/acc.cgi?acc=GSE25307; and (l) Lisowski et al. dataset is available in GEO under the accession number: GSE50567 https://www.ncbi.nlm.nih.gov/geo/query/acc.cgi?acc=GSE50567.
CODE AVAILABILITY
The code and datasets are available in GitHub at the following link: https://github.com/getzlab/pathway_based_biomarker

Field-specific reporting

Please select the one below that is the best fit for your research. If you are not sure, read the appropriate sections before making your selection.

[x] Life sciences [ ] Behavioural & social sciences [ ] Ecological, evolutionary & environmental sciences

For a reference copy of the document with all sections, see nature.com/documents/nr-reporting-summary-flat.pdf

Life sciences study design

All studies must disclose on these points even when the disclosure is negative.

Sample size	We used all tissue types that had more than 20 cell lines. Down sampling analysis showed that in order to achieve saturation in results a higher number of cell lines is needed. We also validated our results in at least two independent datasets.
Data exclusions	Tissue types with less than 20 cell lines were excluded.<br>Eighteen cell lines that were sensitive to more than 20% of the compounds were excluded from the analysis<br>Pathways with low variability across cell lines were excluded.
Replication	All significant results were validated in at least two independent datasets.
Randomization	This is not relevant here.
Blinding	The data was devided into sensitive and not sensitive groups based on AUC z-score levels , no blinded analysis was needed.

Reporting for specific materials, systems and methods

We require information from authors about some types of materials, experimental systems and methods used in many studies. Here, indicate whether each material, system or method listed is relevant to your study. If you are not sure if a list item applies to your research, read the appropriate section before selecting a response.

Materials & experimental systems

n/a	Involved in the study	
[ ]	[x]	Antibodies
[ ]	[x]	Eukaryotic cell lines
[x]	[ ]	Palaeontology
[ ]	[x]	Animals and other organisms
[x]	[ ]	Human research participants
[x]	[ ]	Clinical data

Methods

n/a	Involved in the study	
[x]	[ ]	ChIP-seq
[x]	[ ]	Flow cytometry
[x]	[ ]	MRI-based neuroimaging

Antibodies

Antibodies used	Cleaved Caspase-3 staining (Cell Signaling Antibody (Asp175); 1:1000 dilution) was performed using an automated stainer (BOND RX, Leica Biosystems, Rhenium, Modiin, Israel)
Validation	Immunohistochemistry was performed on 4-µm sections from FFPE tissue samples from ex-vivo organ culture. Hematoxylin and Eosin (H&E) staining was performed using an automated stainer.

Eukaryotic cell lines

Policy information about cell lines

Cell line source(s)	NCI-H211 cell line - ATCC<br>HCC2935, CALU1, NCI-H460, NCI-H1650 - Gifted from Channig Yu (Broad Institute)

Authentication	none of the cell lines used were authenticated
Mycoplasma contamination	All cell lines were tested negative for mycoplasma contamination
Commonly misidentified lines (See ICLAC register)	NA

Animals and other organisms

Policy information about studies involving animals; ARRIVE guidelines recommended for reporting animal research

Laboratory animals	Patient-derived xenografts (PDX) - Jackson Laboratories (TM00204)
Wild animals	NA
Field-collected samples	This study did not involved animals collected from the wild.
Ethics oversight	The Weizmann Institute.

Note that full information on the approval of the study protocol must also be provided in the manuscript.
